# Bioengineered Stroma‐Camouflaged Nanovesicles Disrupt the IDO1/CD276 Immunosuppressive Circuit to Reinvigorate Antitumor Immunity

**DOI:** 10.1002/advs.78142

**Published:** 2026-10-25

**Authors:** Ziqi Zhang, Jing Luo, Mengdi Zhang, Jingying Zhuang, Yating Zheng, Yang Zhang, Zhihong Huang, Xinhua Lin, Shuzhen Xu, Hong Yao, Gang Liu, Bing Chen

**Affiliations:** ^1^ Key Laboratory of Nanomedical Technology (Education Department of Fujian Province) Department of Pharmaceutical Analysis School of Pharmacy Fujian Medical University Fuzhou China; ^2^ Department of Urology Harvard Medical School Brigham and Women's Hospital Boston Massachusetts USA; ^3^ State Key Laboratory of Molecular Vaccinology and Molecular Diagnostics & Center for Molecular Imaging and Translational Medicine School of Public Health Xiamen University Xiamen China; ^4^ Public Technology Service Center Fujian Medical University Fuzhou Fujian China; ^5^ Core Laboratory Fuzhou Hospital of Traditional Chinese Medicine Affiliated to Fujian University of Traditional Chinese Medicine Fuzhou Fujian China

**Keywords:** biomimetic nanovesicles, CD276, immunotherapy resistance, tumor‐associated fibroblasts, tumor microenvironment reprogramming

## Abstract

The cooperative upregulation of IDO1 and the immune checkpoint CD276 in solid tumors establishes a dual immunosuppressive circuit that depletes tryptophan, accumulates kynurenines, and silences T‐cells. We engineered fibroblast‐mimetic nanovesicles (AM@UIO‐BMS/DOX) that uniquely integrate stromal‐camouflaged tumor accumulation, CD276 checkpoint blockade, and co‐delivery of doxorubicin with the clinical IDO1 inhibitor BMS‐986205. The fibroblast‐derived membrane coating promotes accumulation in fibroblast‐rich tumor regions, while surface‐anchored anti‐CD276 scFv enables precision tumor specific targeting and concurrent checkpoint neutralization. Within the tumor microenvironment, doxorubicin elicits immunogenic cell death, and BMS‐986205 metabolically recalibrates the tryptophan–kynurenine equilibrium, relieving the metabolic suppression that otherwise blunts T‐cell priming. In syngeneic tumor models, this bioengineered platform profoundly remodeled the immune microenvironment, as evidenced by a tripartite shift: enhanced dendritic cell maturation and cross‐presentation, expansion of Granzyme B^+^ cytotoxic CD8^+^ T lymphocytes, and selective depletion of CD25^+^FoxP3^+^ regulatory T cells. This concerted action effectively converted immunologically “cold” tumors into an inflamed phenotype, overcoming intrinsic chemoresistance and adaptive immune evasion. Collectively, our findings establish that bioengineered stroma‐camouflaged nanovesicles disrupt the dual IDO1/CD276 immunosuppressive circuit, enhancing antitumor immunity and offering a promising strategy to surmount chemo‐immunotherapy resistance in advanced solid tumors.

## Graphical abstract

Bioengineered stroma‐camouflaged nanovesicles (AM@UIO‐BMS/DOX) convert the immunosuppressive tumor niche into an immunoresponsive milieu by disrupting the IDO1/CD276 circuit. Anti‐CD276 membrane camouflage guides tumor accumulation and deep penetration, enabling controlled co‐delivery of DOX and the IDO1 inhibitor BMS‐986205. DOX triggers immunogenic cell death and dendritic cell maturation, while CD276 blockade and IDO1 inhibition relieve T‐cell suppression and expand cytotoxic T lymphocytes, together reinvigorating antitumor immunity.

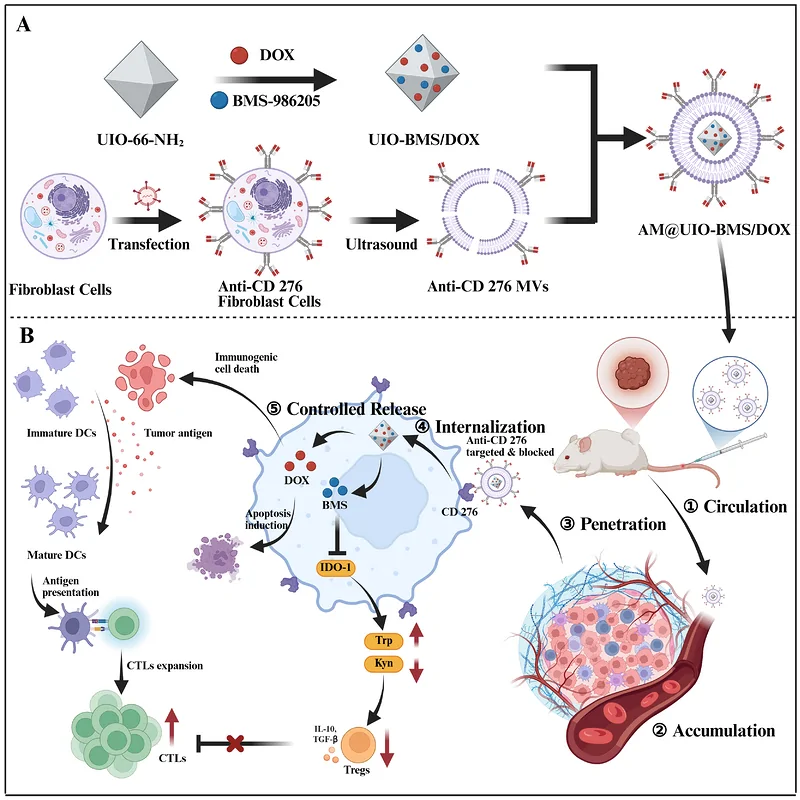

## 1 Introduction

Triple‐negative breast cancer (TNBC), characterized by the absence of estrogen receptor, progesterone receptor, and HER2 amplification, represents the most aggressive and recalcitrant subtype of breast cancer [1, 2, 3]. While the emergence of immune checkpoint blockade (ICB), particularly targeting the PD‐1/PD‐L1 axis, has offered a transformative therapeutic paradigm, clinical benefits remain restricted to a minority of patients [4, 5, 6, 7]. This pervasive immunotherapy resistance observed in TNBC is fundamentally orchestrated by a profoundly immunosuppressive tumor microenvironment (TME) [8, 9]. This cold tumor phenotype is not merely a result of low mutational burden but is an active product of a multifaceted suppressive architecture that employs redundant immunological and metabolic pathways to evade anti‐tumor immunity [10, 11]. The primary challenge, therefore, lies in identifying and concurrently dismantling the non‐redundant pillars of this resistance while overcoming the physical barriers that impede therapeutic infiltration [12, 13].

A critical, yet often overlooked, driver of this resistance is the aberrant expression of CD276 (B7‐H3) [14, 15]. Unlike classical checkpoints, CD276 is markedly overexpressed in TNBC cells, serving as a potent co‐inhibitory ligand that directly blunts T‐cell proliferation and cytokine production [16, 17]. Beyond its primary immunological role, CD276 signaling facilitates tumor invasion, epithelial‐mesenchymal transition (EMT), and chemoresistance via the activation of oncogenic pathways, such as PI3K/AKT and Wnt/β‐catenin [18, 19, 20, 21, 22]. This aberrant signaling contributes to dysfunctional tumor vasculature, fostering a hypoxic, acidic TME that further entrenches immunosuppression and facilitates recurrence [23, 24, 25].

Simultaneously, the ectopic expression of indoleamine 2,3‐dioxygenase 1 (IDO1) by tumor and stromal cells catalyzes tryptophan depletion and the accumulation of immunosuppressive kynurenines [26, 27]. This metabolic reprogramming induces T‐cell anergy and apoptosis while promoting the differentiation and recruitment of regulatory T cells (Tregs), thereby establishing a self‐sustaining tolerogenic niche [28, 29]. Crucially, the concerted action of CD276 and IDO1 establishes dual immunosuppressive axes that may jointly drive T‐cell exhaustion, foster a tolerogenic dendritic cell phenotype, and expand immunosuppressive populations [30, 31]. Furthermore, this milieu blunts chemotherapy‐induced immunogenic cell death (ICD), a key bridge between cytotoxic killing and immune activation, thereby contributing to chemo‐immunotherapy resistance [32, 33, 34]. Current strategies, primarily centered on single‐agent ICB or its combination with chemotherapy, often fail to dismantle this multifaceted immunosuppressive architecture [35]. The persistence of alternative checkpoints like CD276 and metabolic enzymes like IDO1 provides tumors with critical escape routes, limiting response depth and durability [36]. Although CD276‐targeted nanotherapeutics, engineered membrane‐based delivery systems, and IDO1 inhibitor formulations have each shown preclinical promise, they mainly address a single immunosuppressive axis, leaving compensatory escape pathways intact. In contrast, this work develops one integrated platform co‐targeting CD276 and IDO1 within the same construct, enabling concurrent disruption of two non‐overlapping immunosuppressive pillars in the TME, which separate or sequential administration of the individual components would be unlikely to reproduce. Therefore, there is an urgent need for innovative combinatorial approaches capable of concurrently disrupting these non‐overlapping immunosuppressive pillars within the TME.

However, the therapeutic disruption of these axes is severely hindered by the dense, fibrotic stroma characteristic of TNBC, predominantly shaped by tumor‐associated fibroblasts (TAFs) [37, 38]. TAFs are not only abundant stromal components deeply embedded in the TNBC TME but are also active contributors to the immunosuppressive and fibrotic niche that hinders drug delivery and immune cell infiltration [39, 40]. Nevertheless, their innate TME‐homing and stroma‐penetrating properties offer a sophisticated biomimetic opportunity [41, 42]. Inspired by these biological features of TAFs, we hypothesized that engineering nanovesicles with membranes derived from 3T3 fibroblasts, a well‐characterized and readily expandable fibroblast model that shares key stromal homing‐related properties with TAFs, would create a biomimetic “Trojan horse” platform capable of leveraging natural fibroblast homing to overcome stromal barriers and achieve precise, deep‐tissue targeting [43, 44].

To address these integrated challenges, here, we report the design of a fibroblast‐mimetic nanovesicle platform (AM@UIO‐BMS/DOX) for the tumor‐targeted co‐delivery of doxorubicin (DOX) and the clinical‐grade IDO1 inhibitor BMS‐986205. This system is constructed based on a high‐affinity anti‐CD276 single‐chain antibody (scFv) developed by our group [45]. The porous metal‐organic framework UIO‐66‐NH_2_ serves as the core, loading both DOX and BMS‐986205, and is subsequently cloaked with a membrane derived from anti‐CD276 scFv‐engineered fibroblasts (Scheme 1A). This dual‐functional coating confers both active targeting via CD276 recognition and inherent TME‐penetrating ability. Mechanistically, the CD276 scFv‐modified membrane ensures precise tumor‐homing and physical blockade of the CD276 checkpoint, thereby sensitizing the tumor to immunological signals [46]. Upon internalization into tumor cells and activation within the TME, the platform unleashes DOX to induce ICD, which releases tumor antigens and sounds an immune alarm, while BMS‐986205 reverses IDO1‐mediated metabolic exhaustion by restoring tryptophan balance and depleting the Treg population (Scheme 1B). In vivo evaluations demonstrate that this multi‐pronged strategy significantly enhances DC maturation and expands the cytotoxic CD8^+^ T‐cell repertoire characterized by elevated Granzyme B expression, thereby overcoming dual chemo‐immunotherapy resistance and establishing a potent, systemic anti‐tumor effect in preclinical TNBC models [47].

**SCHEME 1:**
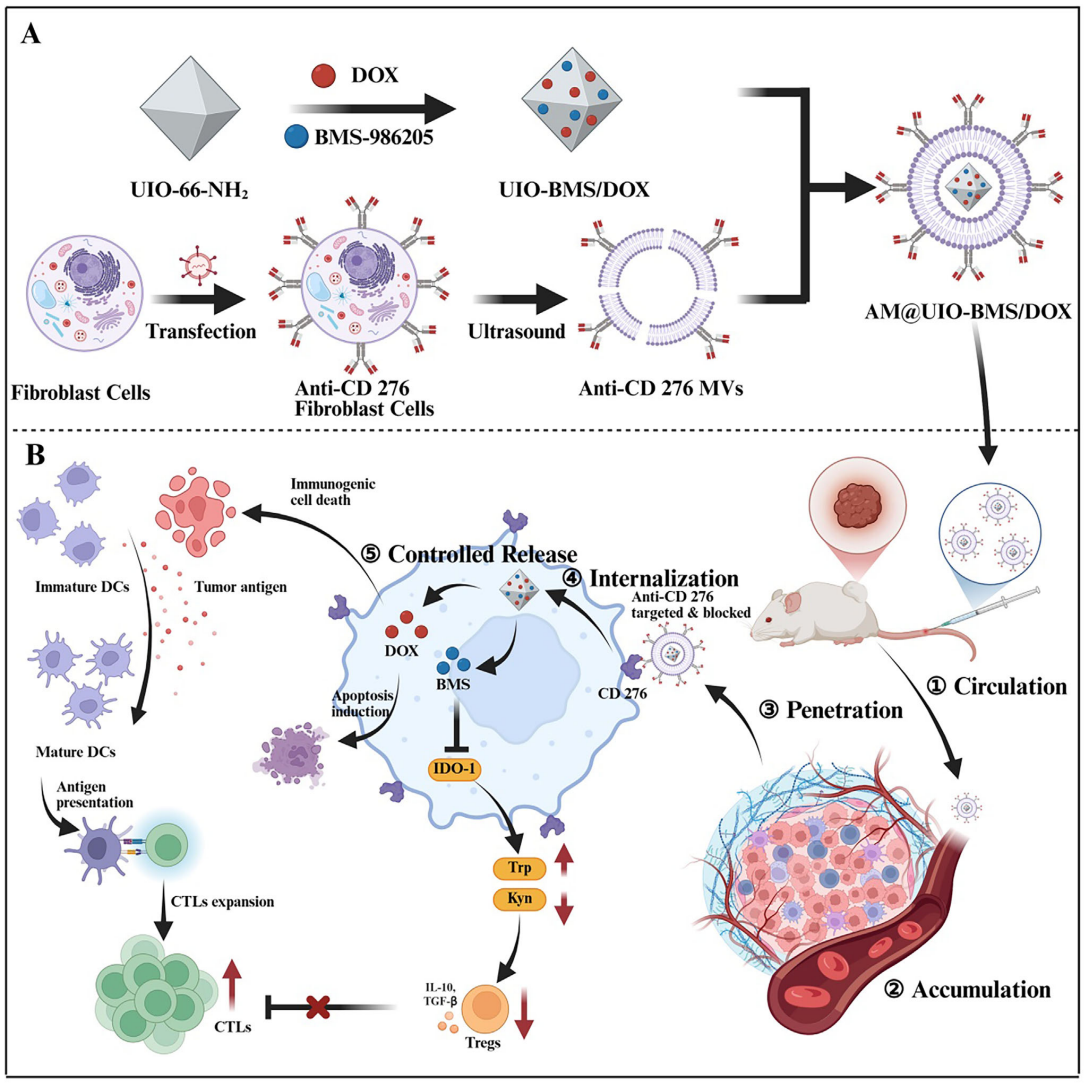
(A) Schematic diagram of anti‐CD276 cell line construction: anti‐CD276 lentivirus was constructed from anti‐CD276 single chain antibody, transfected to construct anti‐CD276 high expression cell line, screened with puromycin, and expanded for culture. And the preparation process of CD276 specific biomimetic nanovesicles. (B) Schematic diagram of the therapeutic mechanism of CD276 specific biomimetic nanovesicles: Chemotherapy drug doxorubicin kills tumor cells, induces ICD effect in tumor cells, activates local immune response at the tumor site, blocks CD276 to release T cell inhibition, enhances anti‐tumor immune response, BMS further downregulates Treg cells, increases tumor immune infiltration, and achieves combined chemotherapy and immune modulation.

## 2 Results and Discussion

### 2.1 Synthesis and Characterization of AM@UIO‐BMS/DOX

UIO‐66‐NH_2_, renowned for its excellent stability, tunable porosity, high specific surface area, and good biocompatibility, serves as an ideal nanocarrier for drug delivery. As depicted in Figure 1A, transmission electron microscopy (TEM) confirmed the synthesis of UIO‐66‐NH_2_ with uniform morphology and crystalline structure. The X‐ray diffraction (XRD) pattern exhibited characteristic peaks at 7.3°, 8.5°, and 25.6°, corresponding to the 110, 200, and 600 planes of UIO‐66‐NH_2_, respectively, which aligns with previously reported data, thus verifying the successful synthesis of phase‐pure material (Figure 1C) [48]. Nitrogen physisorption analysis was employed to evaluate the porous structure. The adsorption‐desorption isotherm (Figure 1D) was classified as Type I (IUPAC), indicative of a microporous structure. The corresponding pore size distribution (Figure 1E) revealed a dominant pore width of approximately 0.50 nm. The Brunauer‐Emmett‐Teller (BET) specific surface area and total pore volume were calculated to be ∼977 m^2^/g and ∼0.450 cm^3^/g, respectively. This substantial surface area and defined microporosity are conducive to facilitate drug loading and to contribute to the sustained release behavior.

**FIGURE 1:**
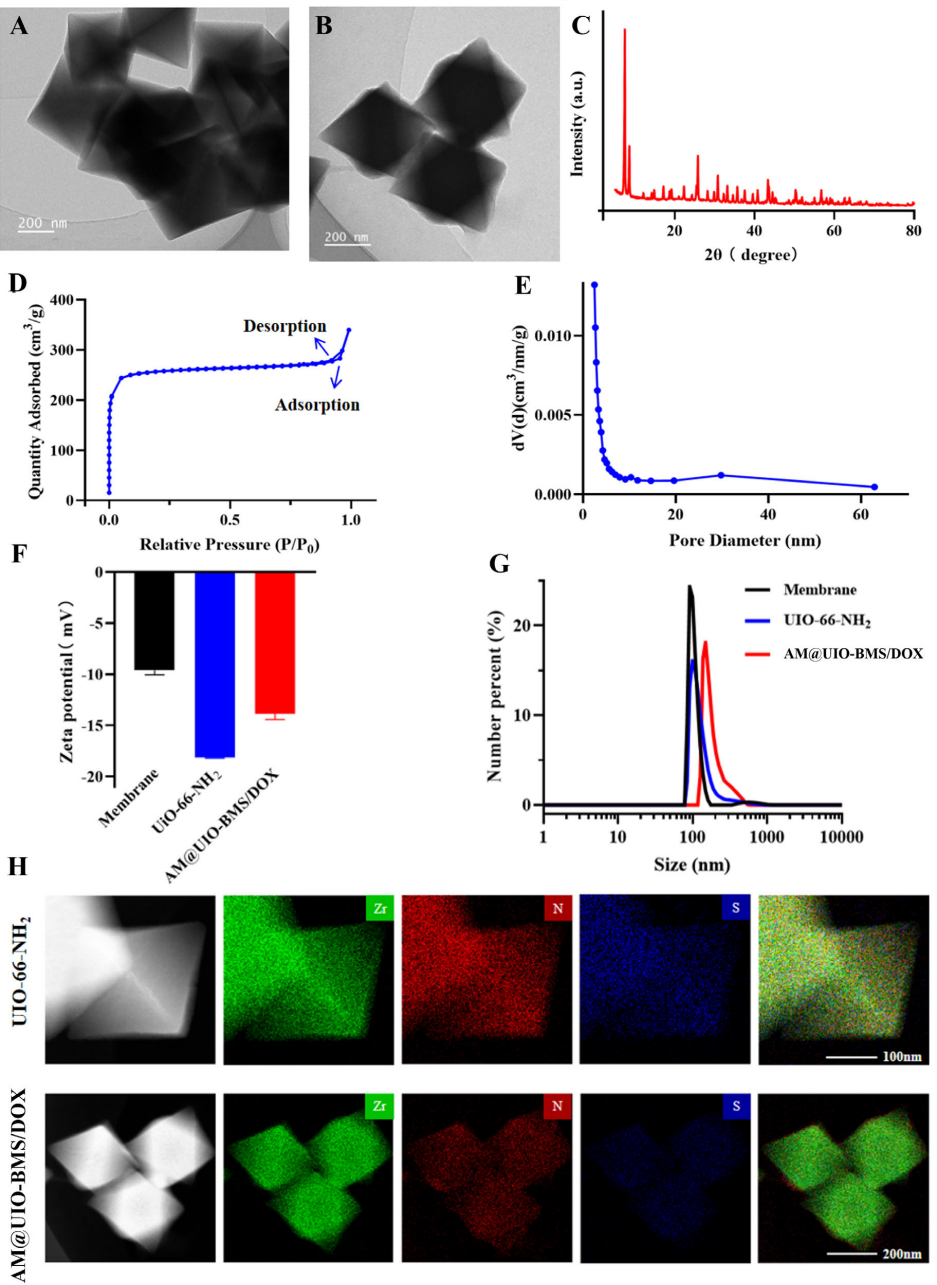
(A) Morphology diagram of UIO‐66‐NH_2_. (B) Transmission electron microscopy (TEM) image of AM@UIO‐BMS/DOX (Scale: 100 nm). (C) X‐ray diffraction (XRD) pattern of UIO‐66‐NH_2_. (D) BET nitrogen adsorption/desorption isotherm of UIO‐66‐NH_2_. (E) BJH pore size distribution of UIO‐66‐NH_2_. (F) Zeta potential distribution diagrams of different nanovesicles. (G) Particle size distribution diagrams of different nanovesicles. (H) Elemental mapping images of UIO‐66‐NH_2_ (Scale: 100 nm) and M@UIO‐66‐NH_2_ (Scale: 200 nm).

Successful encapsulation of the drug‐loaded UIO‐66‐NH_2_ core (UIO@BMS/DOX) within an engineered cell membrane was systematically verified by complementary physicochemical and protein‐based measurements. Dynamic light scattering (DLS) showed that the hydrodynamic diameter increased from ∼176 nm for UIO@BMS/DOX to ∼179 nm for the final AM@UIO‐BMS/DOX (Figure 1G), suggesting the formation of a thin membrane coating. This was corroborated by TEM imaging (Figure 1B), which revealed a translucent outer layer surrounding the UIO@BMS/DOX core. Elemental mapping (Figure 1H) visualized the distributions of Zr, N, and S in the particles, showing a homogeneous distribution of nitrogen (N) across the AM@UIO‐BMS/DOX particles. Zeta potential measurements (Figure 1F) showed that the surface charge shifted from ‐9.62 ± 0.44 mV for bare membrane vesicles to −13.86 ± 0.57 mV for AM@UIO‐BMS/DOX. The more negative surface charge is consistent with the presence of the membrane coating and, based on literature precedent, may contribute to improved stability and reduced non‐specific protein adsorption, potentially favoring prolonged circulation. Together with the DLS size shift, the translucent outer layer observed by TEM, and the membrane‐protein profile detected by SDS‐PAGE, these results support successful assembly of the membrane‐coated formulation.

The source of the coating membrane was a cell line engineered for high expression of anti‐CD276. qPCR analysis (Figure 2A) confirmed a significant upregulation of anti‐CD276 mRNA in the transfected cells compared to the control. Sodium dodecyl sulfate‐polyacrylamide gel electrophoresis (SDS‐PAGE) (Figure 2B) was then used to assess the integrity and successful transfer of membrane proteins during the coating process. The nearly identical protein band patterns among the native cell membrane (Membrane), the engineered membrane (anti‐CD276 Membrane, A‐Membrane), and the membrane‐coated UIO@BMS/DOX core (AM@UIO‐66‐NH_2_) confirmed that the coating procedure preserved the native membrane proteome.

**FIGURE 2:**
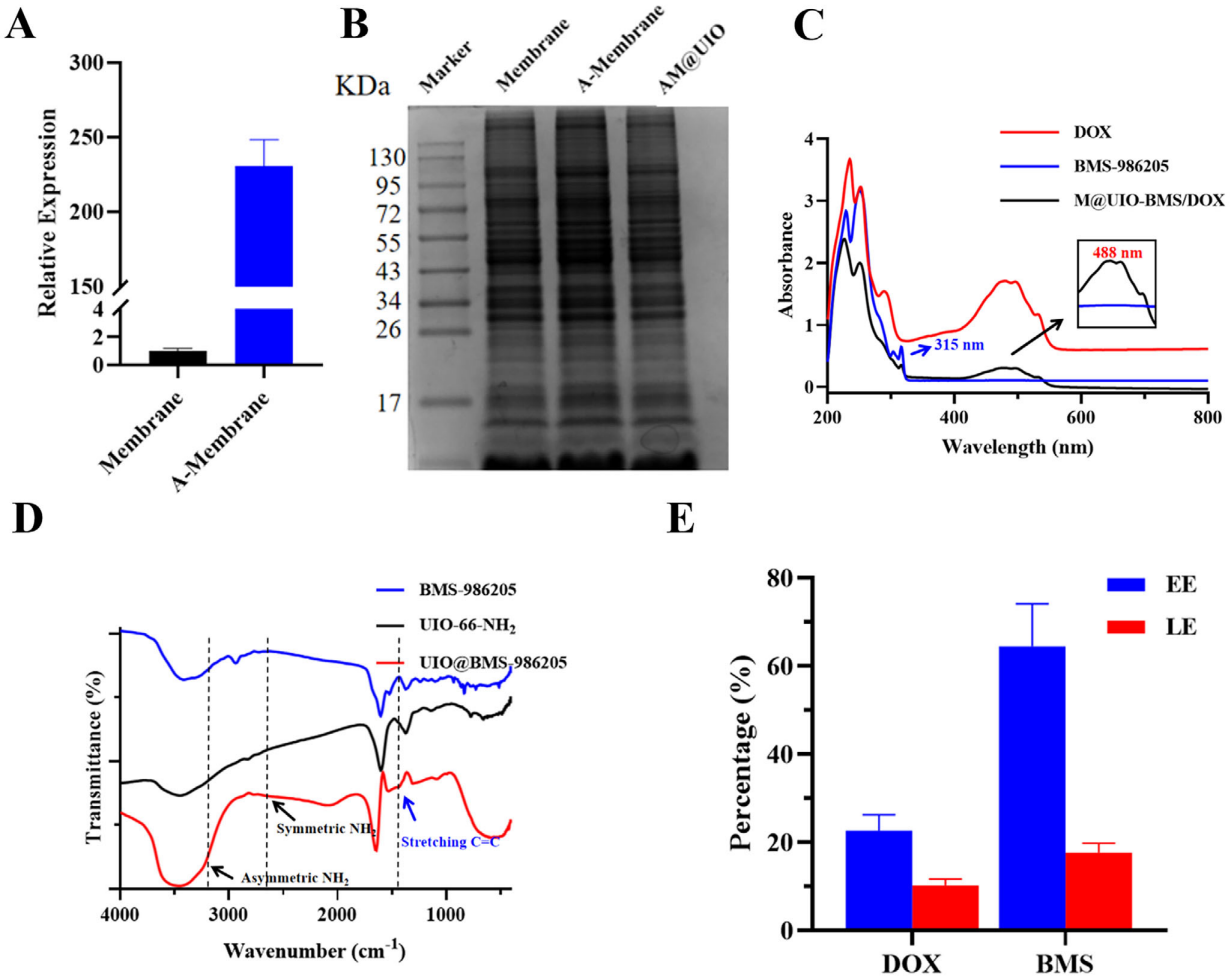
(A) Transcription levels of relevant marker genes in 3T3 cells before and after transfection. (B) Sodium dodecyl sulfate‐polyacrylamide gel electrophoresis (SDS‐PAGE) of 3T3 fibroblast membrane‐derived nanovesicles and engineered nanovesicles (Marker, Nanovesicle, Anti‐CD276 Nanovesicle, and AM@UIO‐66‐NH_2_). (C) UV absorption spectra of DOX, BMS, and M@UIO‐66‐NH_2_. (D) Near‐infrared (NIR) absorption spectra of BMS, UIO‐66‐NH_2_ and UIO@BMS. (E) The encapsulation efficiency (EE) and drug load efficiency (DL) of UIO@BMS/DOX.

Given its high specific surface area and good biocompatibility, UIO‐66‐NH_2_ is an ideal carrier and has been widely used for loading small‐molecule drugs in numerous studies. In this study, DOX and BMS were loaded into the pores of UIO‐66‐NH_2_, and the surface was coated with a cell membrane for in vitro and in vivo anti‐tumor studies. The co‐loading of the chemotherapeutic doxorubicin (DOX) and the immunomodulator BMS‐986205 into the UIO‐66‐NH_2_ framework was validated spectroscopically. UV‐vis spectroscopy (Figure 2C) of the M@UIO‐BMS/DOX displayed the characteristic absorption peaks of DOX at 488 nm and of the quinoline moiety in BMS at 315 nm. Fourier‐transform infrared (FT‐IR) spectroscopy (Figure 2D) provided further evidence, showing the preservation of the framework's characteristic vibrations alongside the appearance of a C = C stretching band attributable to BMS.

Subsequently, high‐performance liquid chromatography (HPLC) was used to establish standard curves for DOX and BMS. The standard curve for DOX was established with DOX concentration as the abscissa (x_1_) and DOX peak area as the ordinate (y_1_), yielding the regression equation y_1_ = 6.209x_1_ + 1.239 (R^2^ = 0.9998), indicating a good linear relationship in the concentration range of 7.8125–250 µg/mL (Figure S1). Similarly, the standard curve for BMS was established with BMS concentration as the abscissa (x_2_) and BMS peak area as the ordinate (y_2_), giving y_2_ = 7.228x_2_ + 16.563 (R^2^ = 0.9998), showing a good linear relationship in the concentration range of 7.8125–250 µg/mL. Based on these standard curves, the encapsulation efficiency (EE) and drug loading capacity (LC) of DOX in UIO@BMS/DOX were calculated (Figure 2E). Notably, BMS exhibited a significantly higher EE (64.44 ± 9.64%) and LC (17.64 ± 2.15%) compared to DOX (EE: 22.63 ± 3.57%; LC: 10.15 ± 1.45%). This disparity can likely be attributed to differences in the molecular size, hydrophobicity, and specific affinity of each drug for the coordinatively unsaturated metal sites and organic linkers within the MOF pores. The successful co‐loading and subsequent membrane coating culminate in the construction of the targeted, multi‐functional AM@UIO‐BMS/DOX nanoplatform.

### 2.2 Assessment of Cellular Uptake and Tryptophan Metabolism Inhibition

To establish an in vitro immune‐stressed model for evaluating CD276‐mediated targeting and IDO1‐mediated metabolic modulation, we first examined the expression of CD276 and IDO1 in 4T1 murine breast cancer cells upon IFN‐γ stimulation. This pretreatment was not intended to fully recapitulate the TNBC tumor microenvironment, but rather to mimic the IFN‐γ‐rich inflammatory milieu that drives the co‐upregulation of these two immunosuppressive molecules and to provide a defined system for assessing targeting specificity and metabolic inhibition. Flow cytometry analysis confirmed that IFN‐γ treatment (50–200 ng/mL) significantly upregulated the surface expression of CD276 on 4T1 cells in a dose‐dependent manner (Figure S2). Western blot analysis further revealed a concomitant marked increase in the expression of the immunosuppressive enzyme IDO1 (Figure 3F and Figure S3), consistent with literature reports that IFN‐γ is a primary inducer of IDO1 in tumors [49]. These results confirm the successful creation of a high‐CD276/high‐IDO1 tumor cell model, which is relevant for evaluating the targeted and combinatorial therapy, while the in vivo therapeutic effects were subsequently assessed in tumor models.

**FIGURE 3:**
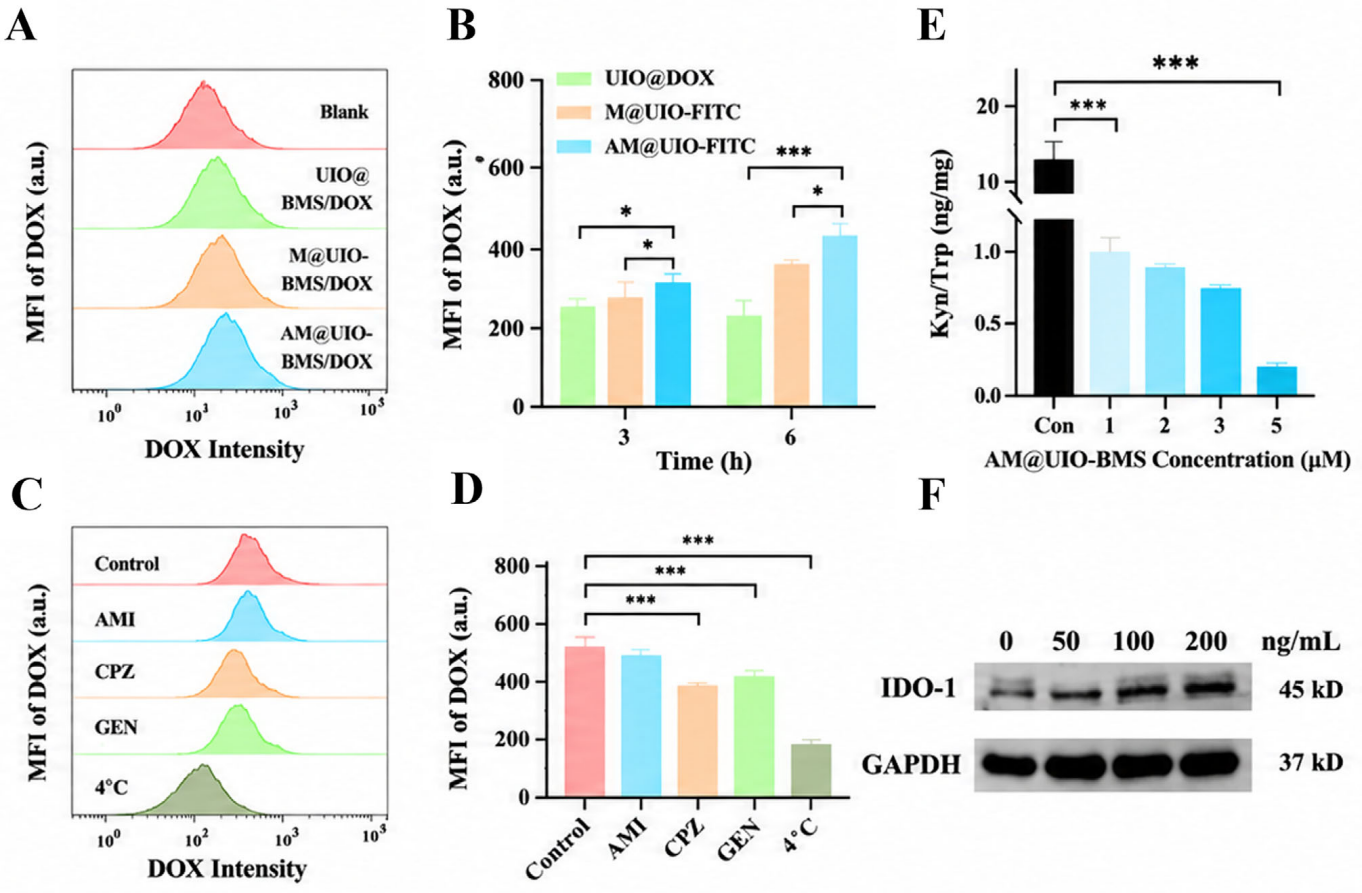
(A) Cell uptake of different nanovesicles at 6 h. (B) DOX fluorescence intensity in cells treated with different nanovesicles at 3 and 6 h. (C) Flow cytometric analysis of DOX fluorescence intensity in 4T1 cells treated with AM@UIO‐BMS under different inhibition conditions. (D) Quantitative column plot showing DOX fluorescence intensity in 4T1 cells treated with AM@UIO‐BMS under different inhibition conditions. (E) Effect of different concentrations of AM@UIO‐BMS on tryptophan metabolism in 4T1 cells. (F) The effect of different concentrations of IFN‐γ (ng/mL) on IDO1 enzyme expression in 4T1 cells.

We next evaluated the cellular internalization efficiency of the nanovesicles using this stimulated model. Flow cytometry analysis of DOX fluorescence showed that cellular uptake was time‐dependent, with significantly higher intensity after 6 h compared to 3 h of co‐incubation (Figure 3A,B). Crucially, AM@UIO‐BMS/DOX exhibited the highest uptake efficiency and the strongest DOX fluorescence signal among all groups at both time points. This superior performance is attributed to the active targeting mediated by the anti‐CD276 antibodies displayed on the biomimetic membrane, which facilitates specific binding and subsequent internalization into CD276‐overexpressing 4T1 cells [50].

To elucidate the endocytic mechanisms of the AM@UIO‐BMS/DOX internalization, a combined pharmacological and biophysical inhibition strategy was employed. Cells were pretreated with specific pathway inhibitors: chlorpromazine (clathrin‐mediated), genistein (caveolae‐mediated), and amiloride (macropinocytosis). Incubation at 4°C was used to inhibit all energy‐dependent processes. Flow cytometric analysis of DOX fluorescence revealed distinct inhibition profiles (Figure 3C,D). Relative to the control (MFI = 540), uptake was most profoundly suppressed at 4°C (MFI = 170), confirming a predominantly energy‐dependent process. Significant inhibition was also observed with chlorpromazine (MFI = 373) and genistein (MFI = 403), indicating concurrent involvement of clathrin‐ and caveolae‐mediated pathways. In contrast, amiloride pretreatment had only a marginal effect (MFI = 500), suggesting a minimal role for macropinocytosis. Collectively, these results demonstrate that cellular entry of AM@UIO‐BMS/DOX occurs primarily via energy‐dependent endocytosis, mediated substantially by both clathrin‐ and caveolae‐dependent mechanisms.

Finally, we assessed the functional efficacy of the loaded IDO1 inhibitor, BMS‐986205. Given the confirmed upregulation of IDO1 by IFN‐γ, we used LC‐MS to measure its impact on tryptophan (Trp) metabolism. After stimulating cells with IFN‐γ and providing exogenous Trp, treatment with AM@UIO‐BMS (at BMS‐equivalent concentrations) for 24 h potently inhibited IDO1 activity. This was evidenced by a dramatic reduction in the Kyn/Trp ratio (Figure 3E). At a BMS concentration of 5 µM, the Kyn/Trp ratio in the AM@UIO‐BMS treated group was only 1/85.26 of that in the control group, demonstrating an exceptionally strong blockade of the immunosuppressive Trp‐Kyn pathway in vitro [51]. This confirms that the encapsulated BMS remains biologically active and can effectively modulate a critical metabolic axis within the TME upon targeted delivery [52].

### 2.3 AM@UIO‐BMS/DOX Facilitates Apoptosis and Immunogenic Cell Death

The pro‐apoptotic efficacy of the nanoplatforms was first assessed by flow cytometry using Annexin V/PI staining (Figure 4B). Quantitative analysis of the apoptotic cell populations (early + late apoptosis) revealed a clear hierarchy in therapeutic potency (Figure S4). Treatment with free BMS‐986205 alone did not induce significant apoptosis compared to the control, consistent with its role as an immunomodulator rather than a direct cytotoxic agent. While free DOX and the non‐targeted nanoparticle UIO@BMS/DOX induced substantial apoptosis, the cell membrane‐coated formulation M@UIO‐BMS/DOX showed enhanced efficacy. Most notably, the targeted nanoparticle AM@UIO‐BMS/DOX elicited the highest level of total apoptosis. This superior performance is attributed to the combined contribution of the biomimetic membrane, which promotes cellular adhesion and uptake, and the anti‐CD276‐mediated active targeting, which significantly enhances specific internalization into CD276‐overexpressing 4T1 cells, thereby maximizing intracellular drug concentration.

**FIGURE 4:**
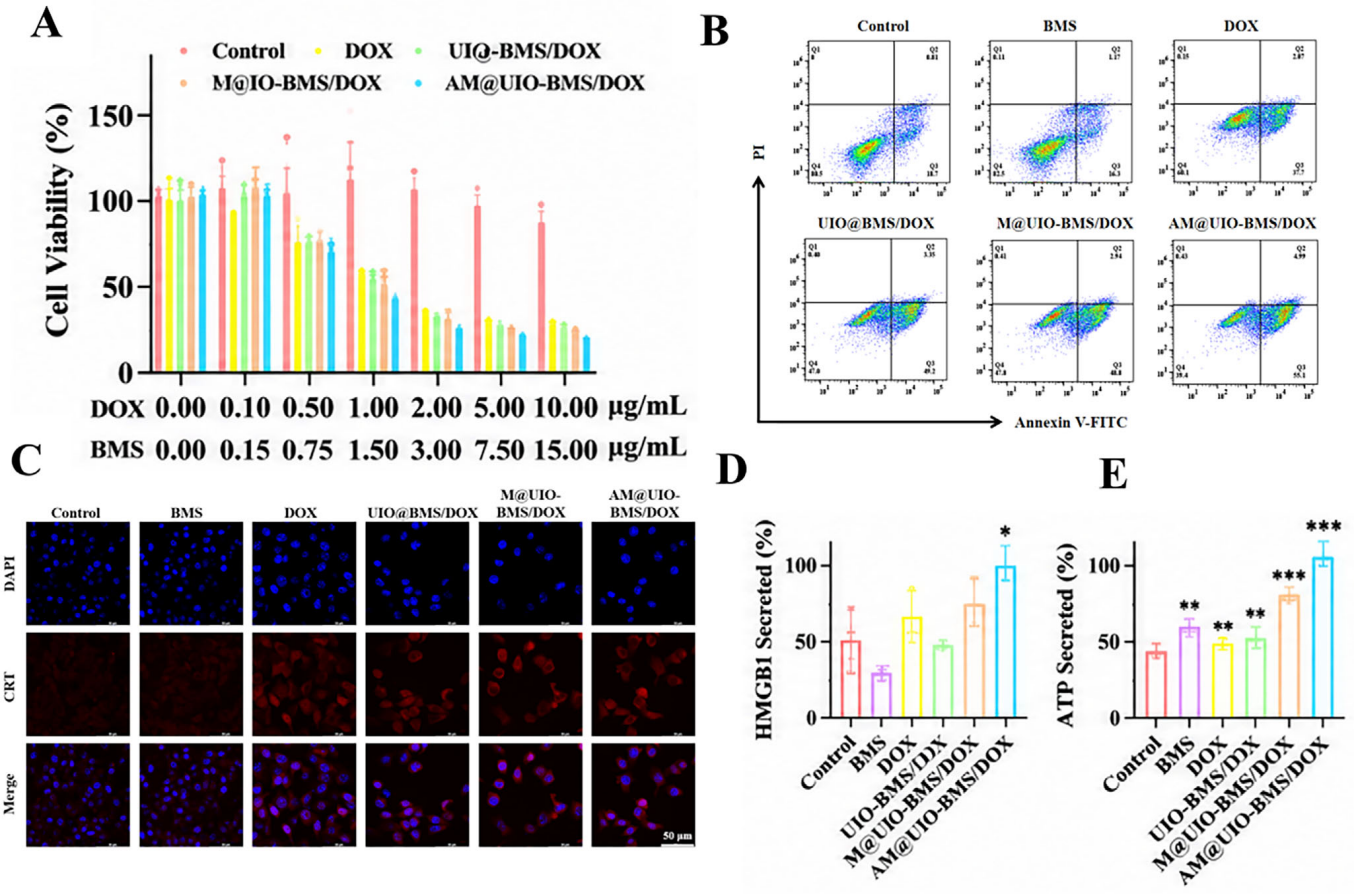
(A) In vitro pharmacological evaluation of 4T1 cells following treatment with various nanovesicles (B) Flow cytometry analysis of 4T1 cells after 24 h of exposure to different nanovesicles. (C) CLSM images showing CRT expression in 4T1 cells treated with different nanovesicles (scale bar: 50 µm). (D) ELISA‐based detection of HMGB1 secretion from 4T1 cells treated with different nanovesicles. (E) ATP release from 4T1 cells treated with different nanovesicles, measured using an ATP detection kit.

Beyond direct cytotoxicity, we investigated the potential of the treatments to induce ICD, a critical process for initiating anti‐tumor immunity. ICD is characterized by the translocation or release of damage‐associated molecular patterns (DAMPs), such as calreticulin (CRT), high‐mobility group box 1 (HMGB1), and ATP, which promote dendritic cell maturation and antigen presentation. Immunofluorescence staining revealed the most pronounced CRT exposure on the surface of cells treated with AM@UIO‐BMS/DOX (Figure 4C). Quantitative assays further confirmed that this group also triggered the highest extracellular release of HMGB1 and ATP (Figure 4D,E). These results collectively demonstrate that AM@UIO‐BMS/DOX is the most potent inducer of ICD among all tested formulations, implying a strong potential to stimulate an adaptive immune response in addition to its direct killing effect.

The in vitro anti‐proliferative activity was quantitatively evaluated using the MTT assay (Figure 4A). Consistent with the apoptosis data, cell viability results confirmed the superior efficacy of the targeted nanoplatform. BMS‐986205 alone showed negligible cytotoxicity even at high concentrations. At equivalent DOX concentrations, UIO@BMS/DOX was more effective than free DOX, underscoring the role of the MOF carrier in improving drug delivery. Coating with the cell membrane (M@UIO‐BMS/DOX) further enhanced cytotoxicity, with the AM@UIO‐BMS/DOX formulation achieving the greatest inhibition of cell proliferation. This pronounced effect directly correlates with its superior cellular uptake, driven by the combined biomimetic and active targeting properties, leading to maximized intracellular drug accumulation and enhanced anti‐tumor activity.

### 2.4 AM@UIO‐BMS/DOX Enables Superior Tumor Accumulation and Biodistribution

To verify the tumor‐targeting capability and pharmacokinetics of our nanoplatform, AM@UIO were labeled with indocyanine green (ICG) and intravenously administered to 4T1 tumor‐bearing BALB/c mice. Real‐time fluorescence imaging revealed time‐dependent tumor accumulation (Figure 5A–E). A clear fluorescent signal was detectable at 9 h post‐injection, which intensified significantly by 24 h. Throughout the imaging period, the AM@UIO‐ICG group exhibited the most pronounced tumor‐specific fluorescence, attributable to the combined contribution of the biomimetic membrane for prolonged circulation and the active targeting conferred by anti‐CD276, which enhances both specific binding and tissue accumulation.

**FIGURE 5:**
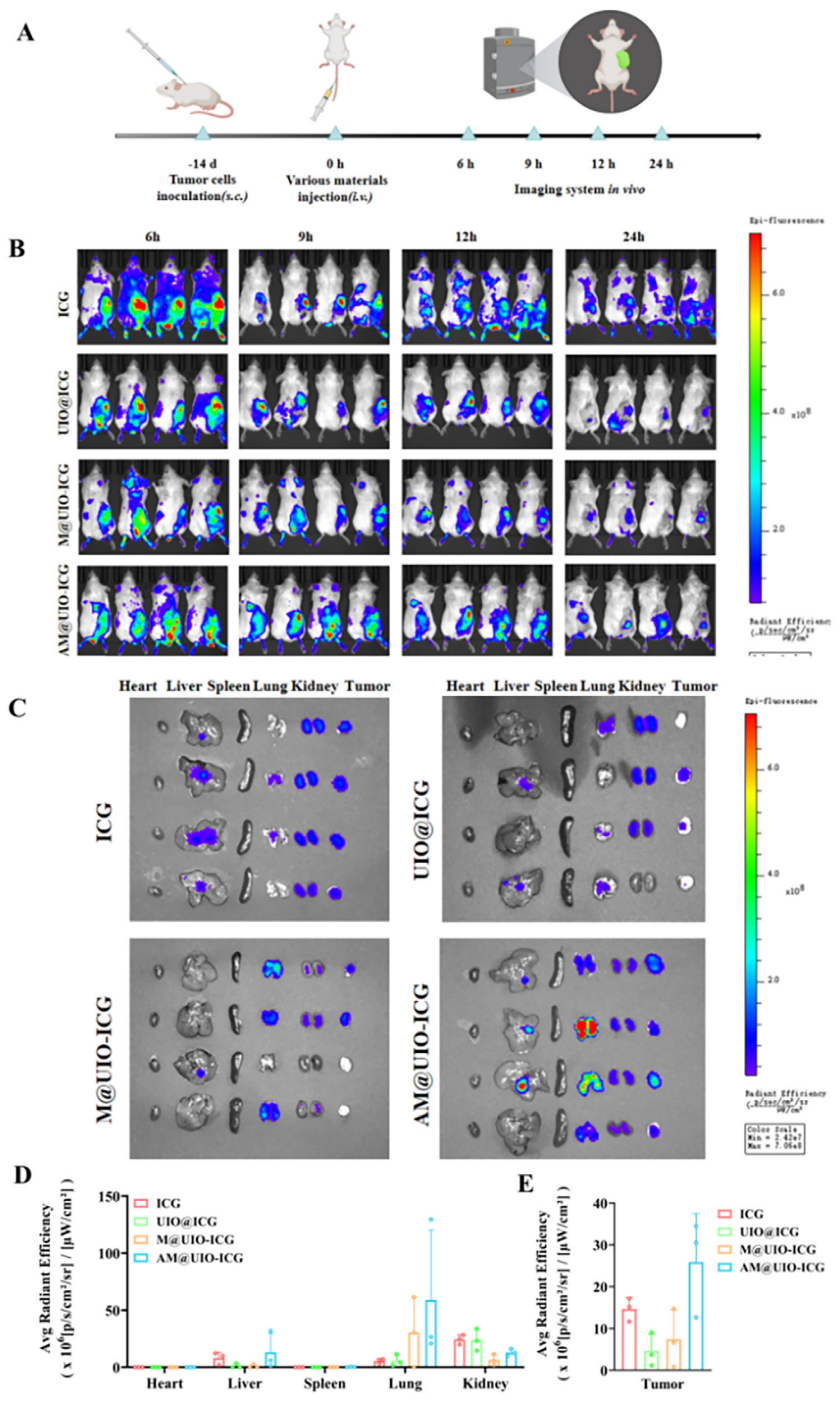
(A) Schematic diagram of the biodistribution study of various treatment groups in 4T1 tumor‐bearing mouse (B) Fluorescence imaging of ICG distribution in mice from different treatment groups at various time points. (C) ICG fluorescence imaging of isolated organs and tumors from different groups of mice at 24 h. (D) Quantitative fluorescence intensity analysis of isolated organs and (E) tumor tissues from different groups of mice at 24 h (n = 4).

### 2.5 AM@UIO‐BMS/DOX Exerts Potent Anti‐Tumor Efficacy With Favorable Systemic Safety

The therapeutic efficacy of AM@UIO‐BMS/DOX was evaluated in a subcutaneous 4T1 breast cancer model. Treatment commenced when tumor volumes reached ∼150 mm^3^, with administrations on days 1 and 4. Tumor growth and body weight were monitored as key indicators of efficacy and systemic toxicity, respectively (Figure 6D,E).

**FIGURE 6:**
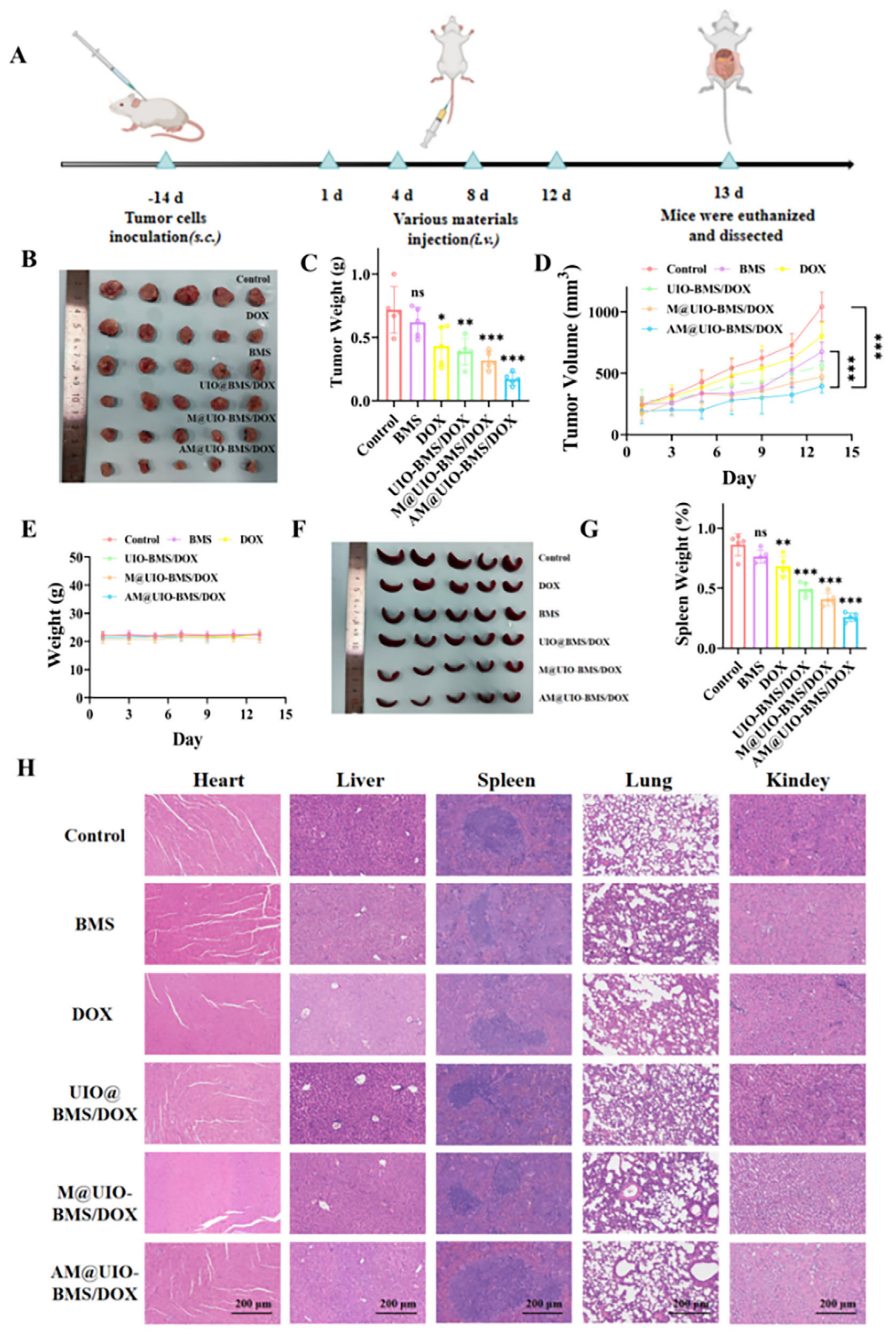
(A) Schematic diagram of the treatment regimen. (B, C) Anatomical tumor mass images and tumor mass weights of 4T1 tumor‐bearing mice treated with different nanovesicles. (D) Tumor volume change trends in 4T1 tumor‐bearing mice treated with different nanovesicles. (E) Body weight change trends of 4T1 tumor‐bearing mice treated with different nanovesicles (n = 5). (F‐G) Spleen images and spleen weights of 4T1 tumor‐bearing mice treated with different nanovesicles (n = 5). (H) H&E staining of various organ tissues in tumor‐bearing mice treated with the Dox group and its related chemotherapy drug groups (scale bar: 200 µm).

The PBS control group exhibited rapid tumor progression. Treatment with free BMS‐986205 alone showed modest growth inhibition, while free DOX provided better but limited suppression. In contrast, UIO@BMS/DOX, M@UIO‐BMS/DOX, and AM@UIO‐BMS/DOX demonstrated significantly enhanced anti‐tumor activity. The targeted formulation AM@UIO‐BMS/DOX achieved the most potent tumor growth inhibition, resulting in the smallest final tumor volumes and weights (Figure 6B,C). This superior efficacy is consistent with its optimized biodistribution and enhanced cellular internalization, leading to maximized local drug delivery and synergistic chemo‐immunotherapy.

Crucially, no significant body weight loss was observed in any group (Figure 6H), indicating minimal systemic toxicity at the administered doses. Furthermore, given that splenomegaly is a hallmark of advanced breast cancer reflecting systemic tumor burden and immune dysregulation, we analyzed spleen size post‐treatment (Figure 6F,G). The control group exhibited significant splenomegaly, whereas spleens from the AM@UIO‐BMS/DOX‐treated mice were the smallest and lightest. This finding provides additional evidence for the potent systemic anti‐tumor effect and potential immune modulation afforded by the targeted chemo‐immunotherapeutic cascade [53]. This finding suggests alleviation of systemic disease burden and is consistent with the observed local tumor control.

### 2.6 Reprogramming the Tumor Immune Microenvironment

To elucidate the immunological mechanisms underlying the in vivo anti‐tumor efficacy, tumor‐infiltrating immune cells were analyzed by flow cytometry after four treatment cycles (Figure 7). Treatment with AM@UIO‐BMS/DOX resulted in a substantial increase in the infiltration of cytotoxic CD8^+^ T lymphocytes into tumors compared to all other groups (Figure 7D,H). This enhanced infiltration was functionally corroborated by a significant elevation in the secretion of Granzyme B (GzmB), a key effector molecule for tumor cell killing (Figure 7B,F and Figure S5). Concurrently, AM@UIO‐BMS/DOX treatment effectively reduced the immunosuppressive tumor microenvironment, as evidenced by a marked downregulation of CD25^+^Foxp3^+^ regulatory T cells (Tregs) within the tumor tissue (Figure 7C,G). Furthermore, analysis of splenic dendritic cells (DCs) revealed that AM@UIO‐BMS/DOX promoted DC maturation, indicated by upregulated expression of the co‐stimulatory molecules CD80 and CD86 (Figure 7A,E). In addition, pulmonary metastasis was assessed at the end of the treatment period. AM@UIO‐BMS/DOX treatment significantly reduced the number of pulmonary metastatic nodules compared with all other groups (Figure 7I,J), consistent with the enhanced systemic anti‐tumor immune response observed in the tumor microenvironment. Collectively, these data demonstrate that AM@UIO‐BMS/DOX orchestrates a comprehensive anti‐tumor immune response by enhancing effector T cell recruitment and function, suppressing Treg‐mediated immunosuppression, and promoting antigen‐presenting cell maturation within the evaluated time frame [54].

**FIGURE 7:**
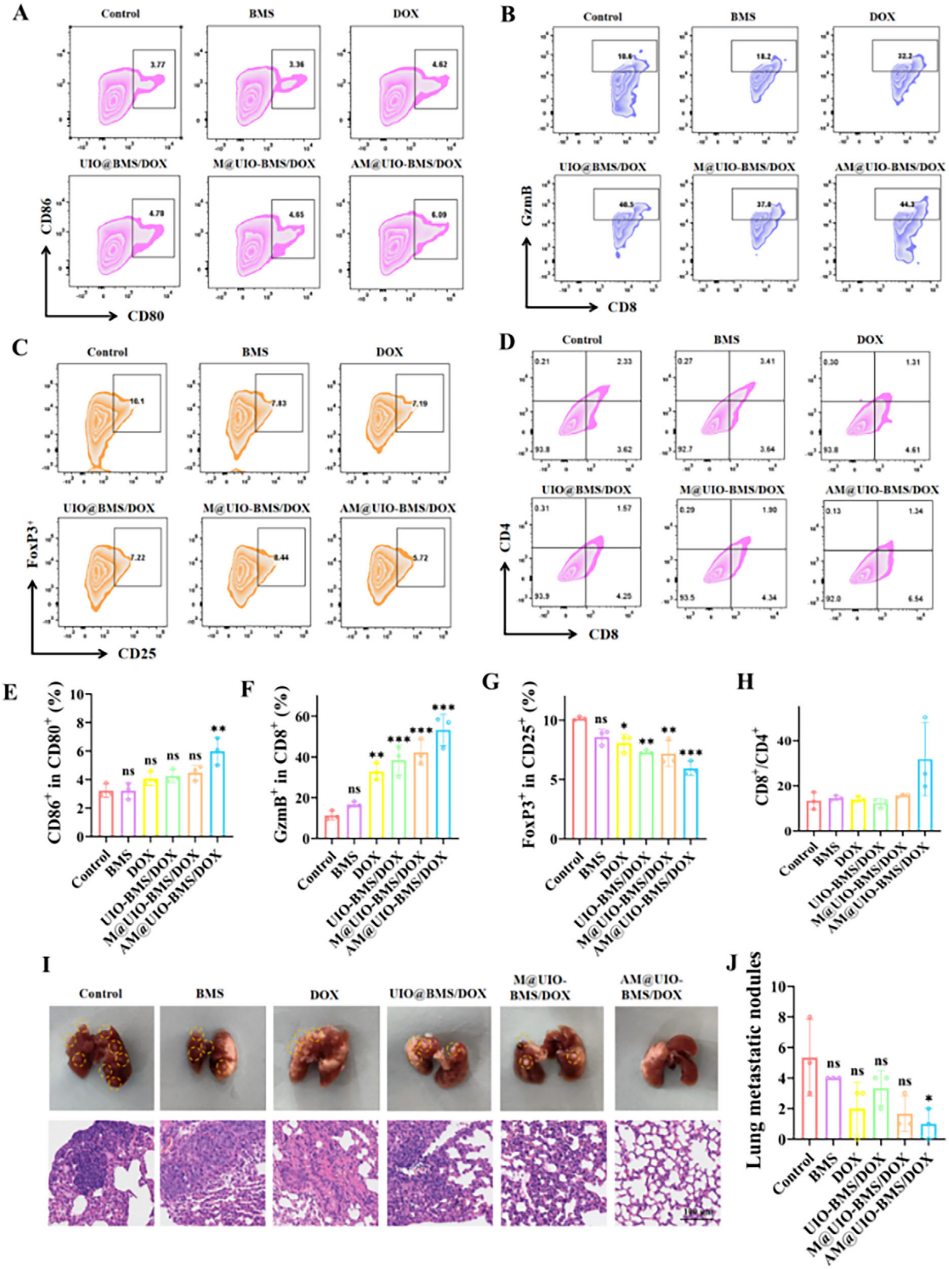
(A, E) Flow‐cytometric analyses of splenic dendritic‐cell maturation based on CD80/CD86 co‐expression, the intratumoral GzmB‐positive fraction within CD8^+^ T cells (B, F), the CD25/FoxP3 double‐positive Treg‐associated population (C, G), and tumor CD4/CD8 distribution with the CD8^+^/CD4^+^ ratio (D, H). (I) Lung metastasis of tumor cells and H&E staining in tumor‐bearing mice after treatment with different nanovesicles (scale bar: 100 µm). (J) Number of lung metastatic nodules in tumor‐bearing mice after treatment with different nanovesicles.

### 2.7 AM@UIO‐BMS/DOX Ensures Systemic Biosafety and Suppresses Metastasis

The in vivo biosafety and therapeutic outcomes of AM@UIO‐BMS/DOX were comprehensively evaluated through histopathological and immunohistochemical analyses. Examination of major organs (heart, liver, spleen, lungs, and kidneys) by H&E staining revealed no detectable pathological lesions in the AM@UIO‐BMS/DOX‐treated group, in stark contrast to the tissue damage (notably cardiotoxicity) commonly associated with DOX clinically (Figure 6H). Serum biochemistry (Figure S6, n = 3) showed ALT, AST, ALB, creatinine (CREA), and uric acid (UA) values within the reference ranges indicated in the original analysis; γ‐glutamyl transferase (γ‐GT) was elevated in the free‐DOX group but remained within the reference range in the AM@UIO‐BMS/DOX group. This confirms the critical role of the targeted nanocarrier in mitigating the systemic toxicities of chemotherapeutic agents. Assessment of excised tumor tissues provided direct evidence of superior therapeutic efficacy. H&E staining demonstrated the most extensive histopathological damage, including nuclear fragmentation and shrinkage, in the AM@UIO‐BMS/DOX group (Figure 8A). This observation was quantitatively supported by the TUNEL assay, which indicated the highest level of apoptosis, and Ki67 immunohistochemistry, which showed the most potent suppression of tumor cell proliferation compared to all other groups, including those treated with free DOX or non‐targeted nanovesicles. Immunofluorescence staining of tumor sections further revealed that the lowest local CD25 signal in the AM@UIO‐BMS/DOX group, which was quantitatively confirmed by CD25 immunohistochemical staining in Figure S7. As CD25 is expressed on both activated conventional T cells and Tregs, this signal was interpreted as a general indicator of local immune activation rather than direct evidence of Treg abundance. These findings collectively demonstrate that the targeted combinatorial nanoplatform induces significantly enhanced tumor cell death while concurrently inhibiting proliferation.

**FIGURE 8:**
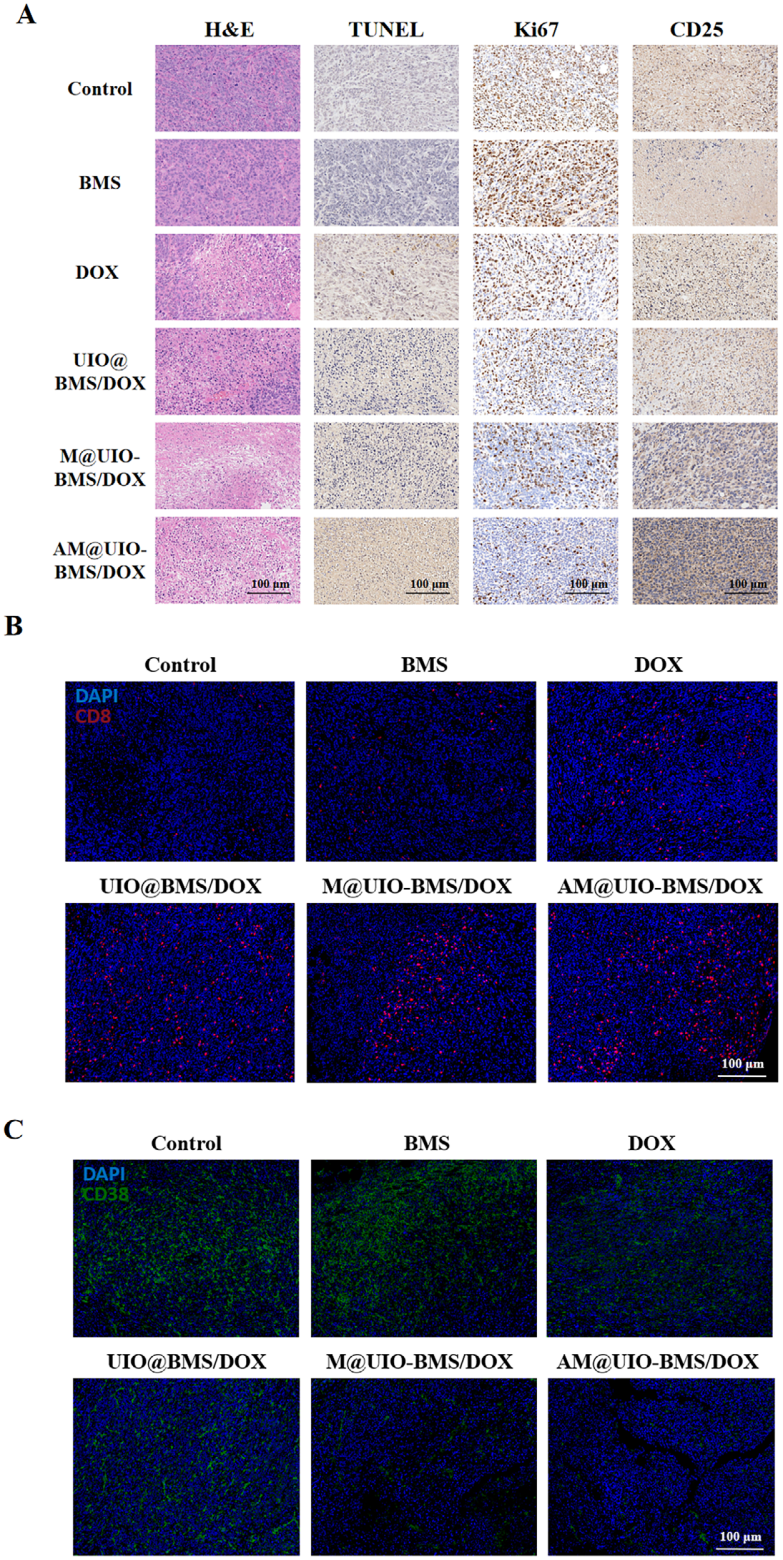
(A) H&E, TUNEL, Ki67 and CD25 staining results of tumor tissue sections from mice treated with different nanovesicles (scale: 100 µm). (B, C) Representative immunofluorescence images of CD8 and CD38 in tumor tissues of tumor‐bearing mice treated with different nanovesicles (immunohistochemical staining, scale bar: 100 µm).

Further analysis of the tumor immune microenvironment revealed that AM@UIO‐BMS/DOX treatment resulted in the greatest infiltration of CD8^+^ T lymphocytes, as visualized by immunofluorescence (Figure 8B,C) and quantitatively supported by the significantly increased CD8 fluorescence intensity shown in Figure S8. Notably, these infiltrating T cells exhibited the lowest co‐expression of CD38, a marker commonly associated with T cell dysfunction or exhaustion in chronic disease contexts. This pattern suggests that the therapy not only promotes T cell recruitment but may also be associated with a less exhausted effector phenotype, which is consistent with reinvigorated antitumor immunity. Nevertheless, the concurrent CD38 reduction and enhanced GzmB production indicate a favorable local immune polarization compared with free DOX treatment, which lacked such immunomodulatory features.

In summary, AM@UIO‐BMS/DOX integrates enhanced tumor‐specific drug delivery, potent direct cytotoxicity, and multifaceted immune activation, resulting in exceptional primary tumor suppression, significant reduction of pulmonary metastatic nodules, and a markedly improved safety profile by circumventing the dose‐limiting toxicities of standard chemotherapy at the evaluated dose schedule [55].

## 3 Conclusions

In summary, we have developed a fibroblast‐mimetic, CD276‐targeted nanoplatform (AM@UIO‐BMS/DOX) for the cascade chemo‐immunotherapy of triple‐negative breast cancer. This system co‐delivers doxorubicin and the IDO1 inhibitor BMS‐986205 specifically to the tumor site. Extensive in vitro and in vivo evaluations confirm its effective CD276‐targeting capability, favorable biosafety, and potent anti‐tumor efficacy. The mechanism of action involves a coordinated dual attack: doxorubicin induces immunogenic cell death to initiate an immune response, while the concurrent release of BMS‐986205 and blockade of CD276 by the surface scFv cooperatively disrupt the pivotal IDO1 and CD276 immunosuppressive axes. This multi‐pronged strategy successfully reprograms the immunosuppressive tumor microenvironment by depleting Tregs, activating dendritic cells, and promoting robust CD8^+^ T cell infiltration, thereby establishing a sustained systemic anti‐tumor immune response within the evaluated treatment window. Our work presents a promising strategy to counteract dual chemo‐immunotherapy resistance in TNBC through targeted co‐delivery and combined immune pathway modulation. We acknowledge that the current study is designed as a short‐term efficacy evaluation and does not address long‐term immune durability, tumor rechallenge, or abscopal effects. Further longitudinal studies are warranted to determine whether the observed early‐stage immune remodeling translates into sustained protective immunity. In addition, systematic release profiling of DOX and BMS‐986205 under multiple physiologically relevant conditions was not performed and warrants further investigation.

## 4 Materials and Methods

### 4.1 Reagents

Chemicals and Reagents: Zirconium tetrachloride (ZrCl_4_, Aladdin, Z1511037), 2‐aminoterephthalic acid (Guangfu Fine Chemical), N,N‐dimethylformamide (DMF, Aladdin, D1521160), methanol (Aladdin, M1521045), anhydrous ethanol (Aladdin, E111992), doxorubicin hydrochloride (DOX·HCl, Aladdin, D409016), BCA protein assay kit (Beyotime, P0398), BMS‐986205 (MCE, HY‐101560), SDS‐PAGE gel preparation kit (Beyotime, P0012A), puromycin (Beyotime, ST551), RPMI‐1640 medium (Procell, PM150110), 0.25% trypsin (Gibco, 25200072), penicillin‐streptomycin solution (Gibco, 15140122), 3‐(4,5‐dimethylthiazol‐2‐yl)‐2,5‐diphenyltetrazolium bromide (MTT, SIGMA, M2003), dimethyl sulfoxide (DMSO, Aladdin, D670381), 4',6‐diamidino‐2‐phenylindole (DAPI, Beyotime, C1002), Annexin V‐FITC apoptosis detection kit (Beyotime, C1062), mouse high mobility group box 1 (HMGB1) ELISA kit (Solarbio, SEKM‐0145), enhanced ATP assay kit (Beyotime, S0027), anti‐GAPDH antibody (CST, 2118T), anti‐IDO1 antibody (CST, 86630T), anti‐calreticulin (CRT) antibody (Bioss, bsm‐52436R), and indocyanine green (ICG, Aladdin, I423619). Fetal bovine serum (FBS) were acquired from Umedium, 3023A, Gezhe Biotechnology Co., Ltd (Hefei, China).

### 4.2 Cell Culture and Animals

Murine breast cancer cell line 4T1 was purchased from Meisen CTCC Cell Technology Co., Ltd. (Zhejiang, China). Cells were cultured in RPMI‐1640 medium supplemented with 1% (w/v) penicillin‐streptomycin and 10% FBS, and maintained in a humidified incubator at 37°C with 5% CO_2_.

Female BALB/c mice (18–22 g) were obtained from Shanghai SLAC Laboratory Animal Co., Ltd. (Shanghai, China). All mice were housed in a specific pathogen‐free (SPF) barrier facility under standard laboratory conditions with a 12 h light/12 h dark cycle, controlled temperature (22 ± 3°C), and humidity (50 ± 10%). Mice had free access to sterilized food and filtered water. Animal health was monitored regularly during the experiment. All animal procedures were performed in strict accordance with the guidelines and regulations of the Institutional Animal Care and Use Committee of Fujian Medical University (Ethics Approval No: FJMU IACUC 2024‐0059).

### 4.3 Preparation and Characterization of UIO‐66‐NH_2_, UIO@BMS/DOX, and AM@UIO‐BMS/DOX

ZrCl_4_ (5 mmoL) and 2‐aminoterephthalic acid (5 mmoL) were added to 30 mL N,N‐dimethylformamide (DMF), followed by 5 mL deionized water. After thorough stirring, the mixture was transferred to a Teflon‐lined autoclave and crystallized at 120°C for 24 h. After the autoclave cooled to room temperature, the resulting yellow gel was washed three times with DMF and methanol, and dried overnight in a constant‐temperature oven at 100°C, respectively.

UIO‐66‐NH_2_ powder (10 mg) synthesized in Section 4.1.1 was dissolved in 5 mL anhydrous ethanol and ultrasonically dispersed for 5 min in a water bath to obtain a UIO‐66‐NH_2_ solution. DOX (5 mg/mL, dissolved in ultrapure water) and BMS‐986205 (3 mg/mL, dissolved in anhydrous ethanol) were added under high‐speed stirring. The mixture was stirred at 1000 rpm for 16 h under ice bath and dark conditions. After stirring, the solution was centrifuged (15000 rpm, 10 min) and washed three times with ultrapure water to obtain a UIO@BMS/DOX solution. Free DOX and BMS‐986205 in the washing solution were collected, and their encapsulation efficiency and drug loading capacity were detected by HPLC.

Logarithmically growing 3T3 cells were added with lentivirus expressing the anti‐CD276 scFv previously screened and coated by the research group. After mixing, the cell culture plate was incubated overnight in a 37°C, 5% CO_2_ incubator. After 12‐16 h of infection, the culture medium containing lentiviral particles was aspirated, and fresh culture medium containing 5% inactivated fetal bovine serum (FBS) was added to the culture plate for further incubation. After 48 h, GFP fluorescence was observed under an inverted fluorescence microscope, and an appropriate concentration of puromycin was added for drug selection. Finally, cells with high fluorescence intensity and high anti‐CD276 expression were sorted by flow cytometry, expanded in culture, and anti‐CD276 engineered cell membrane vesicles were extracted.

Frozen cell membranes (M or AM) were thawed in advance, mixed with UIO‐66‐NH_2_ at a mass ratio of UIO‐66‐NH_2_ to membrane protein (M or AM) of 5:1, vortexed thoroughly, and ultrasonically treated for 5 min in an ice bath. The mixture was centrifuged (4°C, 12000 rpm, 10 min) and washed three times with pre‐cooled PBS. The supernatant was discarded to remove uncoated cell membranes, and the pellet was resuspended in PBS to obtain M@UIO‐BMS/DOX or AM@UIO‐BMS/DOX, which were characterized for particle size, ζ potential, morphology, etc.

For physicochemical characterization, particle size and ζ potential were measured at 25°C using water as the dispersion medium, and each sample was measured in triplicate. TEM was used to assess morphology, elemental mapping was used to examine Zr, N, and S distributions, and membrane‐protein retention was assessed by SDS‐PAGE. For drug loading analysis, DOX and BMS‐986205 standard solutions (7.8125–250 µg/mL) were quantified by HPLC. Free drug (W_free_) in the collected supernatant/washing solution was determined from the calibration curves, and loaded drug was calculated as W_total_—W_free_. Drug loading (DL) and encapsulation efficiency (EE) were calculated as DL (%) = [(W_total_—W_free_)/W_nanodrug_] × 100% and EE (%) = [(W_total_—W_free_)/W_total_] × 100%, respectively, where W_total_ is the total drug input and W_nanodrug_ is the total mass of nanomaterial.

### 4.4 Analysis of IDO1 Enzyme Expression

WB was used to analyze the expression of IDO1 enzyme in 4T1 tumor cells. Specifically, 4T1 cells were seeded in 6‐well plates at a density of 2×10^5^ cells/well and stimulated with 100 ng/mL IFN‐γ for 24 h. Subsequently, tumor cells were collected, lysed with RIPA cell lysis buffer for 5 min, centrifuged at 10000 rpm for 10 min, and the supernatant was collected. Loading Buffer was added, and the mixture was heated at 100°C for 10 min to prepare protein samples. The subsequent steps followed standard WB protocols: each protein sample was diluted to the same concentration, and 10 µL was added to the separating gel layer of SDS‐PAGE gel electrophoresis, with Marker added to both sides. The gel was then placed in an electrophoresis tank, electrophoresis buffer was added, and proteins were separated at an appropriate voltage. After separation, according to the protein Marker and the molecular weight of the target protein, the bands containing the target protein IDO1 enzyme and GAPDH were collected, blocked with blocking buffer for 30 min, and then incubated with the corresponding primary antibody at 4°C overnight. The next day, the primary antibody was discarded, and the membrane was washed with TBST for 10 min, repeated three times. Subsequently, the membrane was incubated with the corresponding HRP‐labeled secondary antibody at room temperature for 2 h. After incubation, the secondary antibody was removed by washing with TBST, and finally, the bands were developed with ECL chromogenic solution and observed with a BioRad imager.

### 4.5 Evaluation of Cellular Uptake of AM@UIO‐BMS/DOX

To investigate the uptake capacity of different nanomaterials at different incubation times, logarithmically growing 4T1 cells were seeded in 6‐well plates at a density of 2×10^5^ cells/well, cultured in RPMI‐1640 containing 10% FBS, and stimulated with IFN‐γ for 24 h. After incubation in a 37°C, 5% CO_2_ incubator, M@UIO‐BMS/DOX and AM@UIO‐BMS/DOX were added to each well, with two time points (3 h and 6 h) and three replicate wells per time point. After incubation, cells were digested and collected, centrifuged at 1000 rpm for 3 min, the supernatant was aspirated, washed with PBS, and passed through a 70 µm cell sieve. The DOX fluorescence intensity was quantitatively analyzed by flow cytometry.

To explore the uptake mechanism of AM@UIO‐BMS/DOX, several common endocytic inhibitors were used for evaluation: chlorpromazine hydrochloride (CPZ, inhibitor of clathrin‐mediated endocytosis), amiloride hydrochloride (AMI, inhibitor of macropinocytosis), genistein (GEN, inhibitor of caveolin‐mediated endocytosis), and a low‐temperature environment (4°C) to investigate the role of energy‐dependent endocytic pathways.

Based on the above principles, logarithmically growing 4T1 cells were seeded in 12‐well plates at a density of 5×10^4^ cells/well and stimulated with IFN‐γ for 24 h. After incubation in a 37°C, 5% CO_2_ incubator, five groups were set up: blank control group (Control), chlorpromazine hydrochloride group (50 µM), genistein group (200 µM), amiloride hydrochloride group (25 µM), and 4°C group. Different solutions were prepared with un‐supplemented RPMI‐1640 or treated at 4°C for 2 h. After incubation, the culture medium was aspirated, and 1 mL AM@UIO‐BMS/DOX diluted with complete RPMI‐1640 culture medium was added to each well, followed by incubation for 3 h. After incubation, cells were washed with PBS, digested, centrifuged, resuspended, collected in flow tubes, and detected by flow cytometry.

### 4.6 Assessment of Tryptophan Metabolism by AM@UIO‐BMS

The IDO1 enzyme catalyzes the conversion of its substrate tryptophan to kynurenine. Therefore, the ratio of tryptophan to its metabolite kynurenine in the supernatant of tumor cells was analyzed to investigate the effect of nanovesicles on the activity of IDO1 enzyme in tumor cells. The specific steps were as follows: 4T1 cells were seeded in 12‐well plates at a density of 5×10^4^ cells/well and stimulated with 100 ng/mL IFN‐γ for 24 h. Subsequently, tryptophan was added exogenously, and tumor cells were treated with different concentrations of AM@UIO‐BMS/DOX (containing different concentrations of BMS). After 24 h, the cell culture medium was collected, and trichloroacetic acid was added to precipitate proteins. The mixture was centrifuged, and the supernatant was collected. The ratio of tryptophan to kynurenine in the supernatant was detected by LC‐MS to analyze the effect of AM@UIO‐BMS on the amino acid metabolism of IDO1 enzyme in 4T1 cells.

### 4.7 Analysis of Apoptosis Induction

During early apoptosis, phosphatidylserine (PS) on the cell membrane translocates from the inner to the outer surface. Annexin V is a Ca^2^^+^‐dependent phospholipid‐binding protein with high affinity for PS, serving as a sensitive probe to detect PS exposed on the cell membrane surface. PS externalization is not unique to apoptotic cells but can also occur in necrotic cells. The difference between the two modes of cell death lies in that the cell membrane remains intact in the initial stage of apoptosis, while the integrity of the cell membrane is disrupted in the early stage of necrosis. Propidium iodide (PI) dye can enter the cell and bind to DNA through the damaged cell membrane. Therefore, a detection method was established using Annexin V binding to the cell membrane surface as an indicator of apoptosis combined with PI dye to detect cell membrane integrity.

### 4.8 Detection of Immunogenic Cell Death Markers

Upon occurrence of ICD in tumor cells, a series of signaling molecules called damage‐associated molecular patterns (DAMPs) are released, mainly including calreticulin (CRT) exposed on the cell surface, high mobility group box 1 (HMGB1) secreted by tumor cells to the extracellular environment, and ATP molecules released by cells. These can be detected by laser scanning confocal microscopy, ELISA kits, and ATP kits, respectively.

### 4.9 Cytotoxicity Assay

The MTT assay was used to investigate the effects of free DOX solution, free BMS solution, UIO@BMS/DOX, M@UIO‐BMS/DOX, and AM@UIO‐BMS/DOX on the viability of 4T1 cells to evaluate the in vitro efficacy of nanovesicles. Logarithmically growing 4T1 cells were seeded in 96‐well cell culture plates at a density of 5×10^3^ cells/well, with 100 µL per well. Zero‐adjustment wells were set, and the edge wells of the 96‐well plate were filled with sterile PBS. After incubation overnight with IFN‐γ in an incubator, seven groups were set up. The final concentrations of DOX added to each well were 0.10, 0.50, 1.00, 2.00, 5.00, and 10.00 µg/mL, and the final concentrations of BMS were 0.15, 0.75, 1.50, 3.00, 7.50, and 15.00 µg/mL, with six parallel wells per group. After incubation in the incubator for 24 h, prepared MTT solution (100 µL, 500 µg/mL) was added, and incubation was continued for 4 h. The culture medium was aspirated, 150 µL DMSO was added to each well, and the plate was oscillated linearly for 15 min. The absorbance (A) at 570 nm was measured using a microplate reader, and the cell viability (%) was calculated.

### 4.10 Biodistribution Analysis of AM@UIO‐BMS/DOX

Logarithmically growing 4T1 cells were digested with trypsin, counted with a hemocytometer, and diluted to a cell concentration of 1.0×10^7^/mL with 1640 medium containing 10% FBS. Each 0.5 mL EP tube was aliquoted with 100 µL and stored on ice for later use. Establishment of mouse breast cancer subcutaneous tumor model: Female BALB/c mice aged 6‐8 weeks were raised in a sterile barrier environment for seven days to adapt to the environment. Hair removal was performed two days in advance, and the prepared 4T1 cells (100 µL/mouse) were injected subcutaneously into the right buttock of each mouse.

In vivo small animal imaging experiments facilitate the intuitive observation of the distribution of nano‐delivery carriers in BALB/c tumor‐bearing mice. The optimal excitation and emission wavelengths of DOX are 488 and 595 nm, respectively, which interfere with the autofluorescence of mice. To monitor the in vivo distribution of nanovesicles, we surface‐labeled them with ICG, a near‐infrared fluorescent dye with optimal excitation and emission at 785 and 810 nm, respectively.

When the tumor volume of the mouse xenograft model reached approximately 150 mm^3^, the mice were randomly divided into 4 groups with 4 mice per group. The four groups of mice were intravenously injected with 100 µL free ICG solution, UIO@ICG, M@UIO‐ICG, and AM@UIO‐ICG nanoparticle solutions (7.5 mg/kg ICG equivalent) via the tail vein, with normal saline as the solvent. At 6 h, 9 h, 12 h, and 24 h after administration, mice were anesthetized with isoflurane, and a small animal in vivo imager was used to observe the tumor tissue accumulation of free ICG solution, UIO@ICG, M@UIO‐ICG, and AM@UIO‐ICG at different times in mice to evaluate the tumor targeting of nanovesicles.

### 4.11 Anti‐Tumor Efficacy of AM@UIO‐BMS/DOX

After establishing the mouse tumor‐bearing model with 4T1 cells, when the tumor size reached approximately 100 mm^3^, the tumor‐bearing mice were randomly divided into 6 groups: Control (PBS), BMS group, DOX group, UIO@BMS/DOX group, M@UIO‐BMS/DOX group, and AM@UIO‐BMS/DOX group, with 5 mice per group. Administration was performed via tail vein injection starting from Day 1 at a dose of 2.5 mg/kg DOX (or 15 mg/kg BMS) equivalent, with four intravenous administrations on Days 1, 4, 8, and 12, consistent with the treatment timeline shown in Figure 6A. Tumor volume and mouse body weight were recorded during the treatment period. Mice were euthanized on Day 13 and dissected to collect tumor tissues and major organs (heart, liver, spleen, lung, kidney). The size changes of tumors and spleens in different groups were measured, and then tumor tissues and major organs were fixed in 4% paraformaldehyde for subsequent H&E staining, TUNEL, IHC, and immunofluorescence analyses to evaluate the therapeutic effect in mice.

### 4.12 Modulation of the Tumor Immune Microenvironment

Tumor blocks (0.04‐1 g) were cut into 2‐4 mm pieces and transferred to gentle MACS C tubes containing 3.5 mL mixed enzyme solution (DNase and collagenase). After brief centrifugation at 300 g to settle the sample tissue to the bottom of the tube, the C tube was tightened, inverted, and installed into the sleeve of the gentle MACS tissue processor (ensuring the sample material was located in the area of the rotor/stator), and the program was run. After the program ended, the C tube was removed from the gentle MACS tissue processor, 13 mL RPMI 1640 was added to each C tube to resuspend the sample, which was then filtered through a 70 µm filter, and the cell suspension was collected in a 50 mL tube. The cell suspension was centrifuged at 500 g for 6 min, the supernatant was quickly discarded, the cell pellet was resuspended, 3 mL 1× red blood cell lysis buffer was added, mixed thoroughly, lysed at room temperature for 12 min, 7 mL D‐PBS was added, and centrifuged at 500 g for 6 min. The supernatant was discarded, the cell pellet was resuspended, 10 mL D‐PBS was added, mixed thoroughly, and the original concentration of cells was counted. Flow cytometry was used to detect the proportion of immune cells in tumor tissues. For the marker‐defined analyses displayed in Figure 7, dendritic‐cell maturation was reported from CD80/CD86 co‐expression, GzmB was reported as the GzmB‐positive fraction within CD8^+^ T cells, the Treg‐associated readout used CD25/FoxP3 co‐expression, and CD4/CD8 distributions were used to calculate the CD8^+^/CD4^+^ ratio.

For flow‐cytometric immune profiling, single‐color tubes were prepared for fluorescence compensation, and FVS780 was used for live/dead discrimination. The documented antibody panel included FITC‐anti‐CD3, BV421‐anti‐CD25, BV650‐anti‐CD86, PerCP/Cy5.5‐anti‐CD8a, BV510‐anti‐CD4, PE‐anti‐FoxP3, and PE/Cy7‐anti‐Granzyme B. Fc receptors were blocked before multicolor staining. FoxP3 and Granzyme B were stained after fixation/permeabilization using BD Perm/Wash buffer. Samples were passed through a 70 µm cell strainer before acquisition, and data were analyzed using FlowJo version 10.0.

### 4.13 Statistical Analysis

Unless otherwise specified, experiments were repeated at least three times and data are presented as mean ± SD. Statistical analyses were performed using GraphPad Prism 8.3.0. Comparisons between two groups were performed using Student's t‐test. Significance was denoted as ns, not significant; ^*^ *p* < 0.05; ^**^ *p* < 0.01; and ^***^ *p* < 0.001.

## Author Contributions

**Ziqi Zhang**: writing – original draft, visualization, validation, formal analysis, data curation. **Jing Luo**: writing – original draft, visualization, validation, methodology. **Mengdi Zhang**: formal analysis, data curation, methodology, investigation. **Jingying Zhuang**: investigation. **Yating Zheng**: methodology. **Yang Zhang**: formal analysis, data curation. **Zhihong Huang**: investigation. **Xinhua Lin**: resources. **Shuzhen Xu**: writing – review and editing, visualization, supervision. **Hong Yao**: supervision, resources, project administration. **Gang Liu**: writing – review and editing, visualization, supervision, resources, funding acquisition. **Bing Chen**: conceptualization, writing – review and editing, visualization, supervision, resources, project administration, funding acquisition.

## Conflicts of Interest

The authors declare no conflicts of interest.

## Supporting information




**Supporting File**: advs78142‐sup‐0001‐SuppMat.docx.

## Data Availability

The data that support the findings of this study are availability from the corresponding author upon reasonable request.

## References

[1] H. Marei , K. Bedair , A. Hasan , et al., “Current Status and Innovative Developments of CAR‐T‐Cell Therapy for the Treatment of Breast Cancer,” Cancer Cell International 25, no. 1 (2025): 3. 10.1186/s12935-024-03615-8 PMC11700463 39755633

[2] S. Wu , A. Ge , X. Deng , L. Liu , and Y. Wang , “Evolving Immunotherapeutic Solutions for Triple‐Negative Breast Carcinoma,” Cancer Treatment Reviews 130 (2024): 102817, 10.1016/j.ctrv.2024.102817. 39154410

[3] X. Zheng , J. Zhuang , X. Peng , Y. Zhang , D. Chen , and B. Chen , “DNA Nanotherapeutics Facilitate Efficient Gynecological Cancers Treatment,” Journal of Controlled Release 388 (2025): 114336, 10.1016/j.jconrel.2025.114336. 41101701

[4] L. Licata , M. Dieci , A. C. De , et al., “Navigating Practical Challenges in Immunotherapy for Metastatic Triple Negative Breast Cancer,” Cancer Treatment Reviews 128 (2024): 102762, 10.1016/j.ctrv.2024.102762. 38776613

[5] M. Kundu , R. Butti , V. Panda , et al., “Modulation of the Tumor Microenvironment and Mechanism of Immunotherapy‐Based Drug Resistance in Breast Cancer,” Molecular Cancer 23, no. 1 (2024): 92, 10.1186/s12943-024-01990-4. PMC11075356 38715072

[6] F. Ye , S. Dewanjee , Y. Li , et al., “Advancements in Clinical Aspects of Targeted Therapy and Immunotherapy in Breast Cancer,” Molecular Cancer 22, no. 1 (2023): 105, 10.1186/s12943-023-01805-y. PMC10324146 37415164

[7] Y. Zhang , H. Chen , H. Mo , et al., “Distinct Cellular Mechanisms Underlie Chemotherapies and PD‐L1 Blockade Combinations in Triple‐Negative Breast Cancer,” Cancer Cell 43, no. 3 (2025): 446–463.e7. 10.1016/j.ccell.2025.01.007 39919737

[8] A. Pandey , S. K. Rai , L. Ma , et al., “Combination Immunotherapy as a Promising Strategy to Overcome Immunotherapy Resistance: From Emergence to Next‐Generation Approaches,” Advanced Science 13, no. 35 (2026): 18960, 10.1002/advs.202518960. PMC13292182 41740058

[9] V. Debien , C. A. De , X. Wang , et al., “Immunotherapy in Breast Cancer: An Overview of Current Strategies and Perspectives,” NPJ Breast Cancer 9, no. 1 (2023): 7, 10.1038/s41523-023-00508-3. PMC9925769 36781869

[10] B. Chen , Y. Shi , Y. Zhang , et al., “Four‐Pronged Nanomotor Strategy Achieves Efficient TNBC Treatment via Synergistic Chemo‐Photothermal‐Immune Therapy,” Advanced Functional Materials 35, no. 48 (2025): 2504621, 10.1002/adfm.202504621.

[11] S. Ning , P. Shangguan , X. Zhu , et al., “Pyridinium Rotor Strategy Toward a Robust Photothermal Agent for STING Activation and Multimodal Image‐Guided Immunotherapy for Triple‐Negative Breast Cancer,” The Journal of the American Chemical Society 147, no. 9 (2025): 7433–7444. 10.1021/jacs.4c15534 PMC11887044 39977833

[12] H. Sabit , A. Adel , M. Abdelfattah , et al., “The Role of Tumor Microenvironment and Immune Cell Crosstalk in Triple‐Negative Breast Cancer (TNBC): Emerging Therapeutic Opportunities,” Cancer Letters 628 (2025): 217865, 10.1016/j.canlet.2025.217865. 40516902

[13] F. Xia , Y. Lu , Z. Gong , et al., “Cancer Immunotherapy Based on the Bidirectional Reprogramming of the Tumor Microenvironment by a “Brakes Off/Step on the Accelerator” Core‐Shell Manganese Phosphate/siPD‐L1 Modulator,” Exploration 5, no. 3 (2025): 270009. 10.1002/EXP.70009 PMC12199384 40585771

[14] Y. Guo , X. Wang , C. Zhang , et al., “Tumor Immunotherapy Targeting B7‐H3: From Mechanisms to Clinical Applications,” ImmunoTargets and Therapy 14 (2025): 291–320. 10.2147/ITT.S507522 PMC11960814 40171330

[15] J. Li , H. Liu , Z. Li , et al., “CD276‐Directed Supramolecular Nanoplatform With pH‐Triggered Gemcitabine Release for Potent Tumor Stromal and Vascular Suppression,” International Journal of Pharmaceutics: X 10 (2025): 100452. 10.1016/j.ijpx.2025.100452 PMC12681842 41362439

[16] F. Howard , D. Villamar , G. He , A. Pearson , and R. Nanda , “The Emerging Role of Immune Checkpoint Inhibitors for the Treatment of Breast Cancer,” Expert Opinion on Investigational Drugs 31, no. 6 (2022): 531–548, 10.1080/13543784.2022.1986002. PMC8995399 34569400

[17] J. Pan , R. Chang , M. Zhang , et al., “Targeting WDR12 Unleashes T‐Cell‐Mediated Antitumor Activity in Melanoma by Destabilizing CD276,” Advanced Science 13, no. 52 (2026): 76255, 10.1002/advs.76255. PMC13337114 42363820

[18] T. Deng , Y. Deng , S. Tsao , et al., “Rapidly‐Manufactured CD276 CAR‐T Cells Exhibit Enhanced Persistence and Efficacy in Pancreatic Cancer,” Journal of Translational Medicine 22, no. 1 (2024): 633, 10.1186/s12967-024-05462-7. PMC11229349 38978106

[19] C. Wang , Y. Li , L. Jia , et al., “CD276 Expression Enables Squamous Cell Carcinoma Stem Cells to Evade Immune Surveillance,” Cell Stem Cell 28, no. 9 (2021): 1597–1613.e7. 10.1016/j.stem.2021.04.011 PMC8419062 33945793

[20] L. Li , S. Nian , Q. Liu , et al., “Fully Human Anti‐B7‐H3 Recombinant Antibodies Inhibited Tumor Growth by Increasing T cell Infiltration,” International Immunopharmacology 132 (2024): 111926, 10.1016/j.intimp.2024.111926. 38552297

[21] I. Hagelstein , L. Wessling , A. Rochwarger , et al., “Targeting CD276 for T Cell‐Based Immunotherapy of Breast Cancer,” Journal of Translational Medicine 22, no. 1 (2024): 902, 10.1186/s12967-024-05689-4. PMC11452943 39367484

[22] J. Wang , K. Ahmed , D. Mao , et al., “A Cancer Stem Cell‐Directed Nano‐Chemotherapy Enhances Anti‐PD‐L1 Immunotherapy in Breast Cancer by Inducing Immunogenic Cell Death,” Acta Biomaterialia 218 (2026): 525–541, 10.1016/j.actbio.2026.06.007. 42248285

[23] B. Liu , M. Lv , Y. Duan , et al., “Genetically Engineered CD276‐Anchoring Biomimetic Nanovesicles Target Senescent Escaped Tumor Cells to Overcome Chemoresistant and Immunosuppressive Breast Cancer,” Biomaterials 313 (2025): 122796, 10.1016/j.biomaterials.2024.122796. 39226654

[24] M. J. Khaw , N. A. Zorko , P. R. Kennedy , et al., “Novel Trispecific Killer Engager Targeting B7‐H3 Enhances Natural Killer Cell Antitumor Activity Against Head and Neck Cancer,” Journal for ImmunoTherapy of Cancer 13, no. 7 (2025): 011370, 10.1136/jitc-2024-011370. PMC12306285 40707133

[25] W. Chen , L. Shen , J. Jiang , et al., “Antiangiogenic Therapy Reverses the Immunosuppressive Breast Cancer Microenvironment,” Biomarker Research 9, no. 1 (2021): 59, 10.1186/s40364-021-00312-w. PMC8296533 34294146

[26] B. Zhu , Y. Cai , L. Zhou , et al., “Injectable Supramolecular Hydrogel Co‐Loading Abemaciclib/NLG919 for Neoadjuvant Immunotherapy of Triple‐Negative Breast Cancer,” Nature Communications 16, no. 1 (2025): 687, 10.1038/s41467-025-55904-z. PMC11735626 39814714

[27] J. Luke , K. Gelmon , L. Siu , et al., “Phase 1/2 Study of the Indoleamine 2,3‐Dioxygenase 1 Inhibitor Linrodostat Mesylate Combined With Nivolumab or Nivolumab and Ipilimumab in Advanced Solid Tumors or Hematologic Malignancies,” Clinical Cancer Research 31, no. 11 (2025): 2134–2144, 10.1158/1078-0432.CCR-24-0439. 39670852

[28] M. Wang , Y. Liu , Y. Li , et al., “Tumor Microenvironment‐Responsive Nanoparticles Enhance IDO1 Blockade Immunotherapy by Remodeling Metabolic Immunosuppression,” Advanced Science 12, no. 5 (2025): 2405845, 10.1002/advs.202405845. PMC11791960 39661740

[29] S. Fattori , H. Roux , E. Connen , et al., “Therapeutic Targeting of Tumor‐Infiltrating Regulatory T Cells in Breast Cancer,” Cancer Research 82, no. 21 (2022): 3868–3879, 10.1158/0008-5472.CAN-22-0654. 36040356

[30] Y. Fujiwara , S. Kato , M. Nesline , et al., “Indoleamine 2,3‐Dioxygenase (IDO) Inhibitors and Cancer Immunotherapy,” Cancer Treatment Reviews 110 (2022): 102461, 10.1016/j.ctrv.2022.102461. PMC12187009 36058143

[31] T. Li , Y. Wang , J. Zhu , et al., “Matrix‐Modulating pH‐Gated Hydrogel for Coordinated Regulation of Cancer‐Associated Fibroblast Phenotype and Mn^2^ ^+^‐STING Activation for Enhanced Tumor Immunotherapy,” Acta Biomaterialia 212 (2026): 655–670, 10.1016/j.actbio.2026.01.014. 41519360

[32] S. Bai , Z. Lu , Y. Jiang , et al., “Nanotransferrin‐Based Programmable Catalysis Mediates Three‐Pronged Induction of Oxidative Stress to Enhance Cancer Immunotherapy,” ACS Nano 16, no. 1 (2022): 997–1012, 10.1021/acsnano.1c08619. 34928122

[33] J. Li , K. Zheng , L. Lin , et al., “Reprogramming the Tumor Immune Microenvironment Through Activatable Photothermal Therapy and GSH depletion Using Liposomal Gold Nanocages to Potentiate Anti‐Metastatic Immunotherapy,” Small 20, no. 51 (2024): 2407388. 10.1002/smll.202407388 39359043

[34] L. Li , B. Wang , Q. Li , et al., “A TCR Nanovesicle Antibody for Redirecting T Cells and Reversing Immunosuppression as a Tumor Immunotherapy Strategy,” Journal of Controlled Release 384 (2025): 113869, 10.1016/j.jconrel.2025.113869. 40412660

[35] X. Miao , B. Sun , J. Zhang , et al., “In Vivo Analysis Techniques for Antibody Drug: Recent Advances and Methodological Insights,” Journal of Pharmaceutical Analysis 15, no. 12 (2025): 101314, 10.1016/j.jpha.2025.101314. PMC12765256 41492456

[36] Y. Xia , L. Hu , Y. Hu , et al., “Co‐Delivery of a STING Agonist and Indoleamine 2,3‐Dioxygenase 1 Blockade Activates Type I Dendritic Cells in Cancer,” Journal of Controlled Release 392 (2026): 114731, 10.1016/j.jconrel.2026.114731. 41702511

[37] Q. Chen , Y. Zhou , X. Zhu , et al., “Enhancing PD‐1 Inhibitor Efficacy in Cholangiocarcinoma by Reprogramming Tumor‐Associated Neutrophils With CAF‐Derived Extracellular Vesicles Loaded With Ruxolitinib,” Advanced Functional Materials 35, no. 45 (2025): 2423217, 10.1002/adfm.202423217.

[38] Z. Guo , X. Li , L. Huang , Y. Yan , M. Gao , and J. Huang , “Pan‐Cancer Single‐Cell and Spatial Transcriptomics Implicate Cancer‐Associated Fibroblasts in Neutrophil Immunosuppressive Phenotypic Transitions and Immunotherapy Resistance,” Functional & Integrative Genomics 25, no. 1 (2025): 204, 10.1007/s10142-025-01706-x. 41068349

[39] J. Cumming , P. Maneshi , M. Dongre , et al., “Dissecting FAP+ Cell Diversity in Pancreatic Cancer Uncovers an Interferon‐Response Subtype of Cancer‐Associated Fibroblasts With Tumor‐Restraining Properties,” Cancer Research 85, no. 13 (2025): 2388–2411, 10.1158/0008-5472.CAN-23-3252. PMC12214878 40215177

[40] H. Cho , M. Kwon , and J. Varner , “Targeting Tumor‐Associated Macrophages and Cancer‐Associated Fibroblasts to Overcome Therapeutic Resistance in Hepatocellular Carcinoma,” Clinical Cancer Research 32, no. 7 (2026): 1196–1209, 10.1158/1078-0432.CCR-25-2944. PMC12875650 41636792

[41] W. Yi , S. Hu , X. Qian , W. Yan , and Y. Li , “Synthetic Biology‐Based Engineering Cells for Drug Delivery,” Exploration 5, no. 2 (2025): 20240095. 10.1002/EXP.20240095 PMC12087405 40395752

[42] Y. Meng , C. Chen , R. Lin , et al., “Mitochondria‐Targeting Virus‐Like Gold Nanoparticles Enhance Chemophototherapeutic Efficacy Against Pancreatic Cancer in a Xenograft Mouse Model,” International Journal of Nanomedicine 19 (2024): 14059–14074, 10.2147/IJN.S497346. PMC11693971 39748900

[43] Z. Chen , Y. Hu , X. Yue , et al., “Matrix‐Bound Nanovesicles Isolated From Decellularized Tumors as Platforms for Targeting Parent Tumor Cells and Tumor‐Associated Stromal Cells,” Materials Today Bio 35 (2025): 102355. 10.1016/j.mtbio.2025.102355 PMC12513100 41080737

[44] F. Meng , C. Zhang , Y. Ma , et al., “Harnessing Chlorella Vulgaris‐Derived Extracellular Vesicles for Potentiated Cancer Immunotherapy,” Biomaterials 336 (2026): 124442, 10.1016/j.biomaterials.2026.124442. 42442284

[45] Y. Qiu , Z. Wang , L. He , et al., “A Novel Proteolysis‐Targeting Chimera Strategy Targeting Multiple Immune Checkpoints Containing ITIMs Enhances Antitumor Immunity,” Journal of Pharmaceutical Analysis 16, no. 3 (2026): 101511, 10.1016/j.jpha.2025.101511. PMC13015236 41890822

[46] B. Chen , K. Zheng , S. Fang , et al., “B7H3 Targeting Gold Nanocage pH‐Sensitive Conjugates for Precise and Synergistic Chemo‐Photothermal Therapy Against NSCLC,” Journal of Nanobiotechnology 21, no. 1 (2023): 378, 10.1186/s12951-023-02078-9. PMC10583352 37848956

[47] Z. Yang , T. Lan , R. Diao , et al., “CD^4+^ Th Cell‐Derived Extracellular Vesicles Orchestrate CD^8+^ T Cells and Eosinophils to Enhance Anti‐Tumor Immunity,” Small 22, no. 36 (2026): 13605. 10.1002/smll.202513605 42125790

[48] Y. Yang , Q. Liu , M. Pan , et al., “Genetically Engineered Cellular Membrane‐Camouflaged Nanoparticles Amplify Immune Response Against Recurrent Metastatic Triple‐Negative Breast Cancer,” Biomaterials 330 (2026): 124026, 10.1016/j.biomaterials.2026.124026. 41633299

[49] C. Zhao , Y. Pan , L. Liu , et al., “Hybrid Cellular Nanovesicles Block PD‐L1 Signal and Repolarize M2 Macrophages for Cancer Immunotherapy,” Small 20, no. 31 (2024): 2311702. 10.1002/smll.202311702 38456371

[50] D. Chen , X. Zheng , Q. Gao , B. Chen , and Y. Sun , “Manganese Biomineralized Ferritin Nanoplatforms With Shielding and Stimuli‐Responsive Release for Potentiated Ferroptosis and Multimodal Ovarian Cancer Therapy,” International Journal of Nanomedicine 21 (2026): 548411. 10.2147/IJN.S548411 PMC13036247 41924452

[51] Q. Shi , N. Gong , J. Wang , et al., “Prodrug‐Tethered Lipid Nanoparticles for Synergistic Messenger RNA Cancer Immunotherapy,” Nature Nanotechnology 21, no. 3 (2026): 430–442, 10.1038/s41565-025-02102-z. 41851499

[52] L. Wang , S. Ge , Y. Zhang , et al., “The Crucial Function of IDO1 in Pulmonary Fibrosis: From the Perspective of Mitochondrial Fusion in Lung Fibroblasts and Targeted Molecular Inhibition,” Acta Pharmaceutica Sinica B 15, no. 6 (2025): 3125–3148, 10.1016/j.apsb.2025.04.027. PMC12254770 40654366

[53] Q. Lin , Y. Jing , C. Yan , et al., “Design and Application of pH‐Responsive Liposomes for Site‐Specific Delivery of Cytotoxin From Cobra Venom,” International Journal of Nanomedicine 19 (2024): 5381–5395, 10.2147/IJN.S461728. PMC11164093 38859950

[54] Q. Li , Y. Ding , Y. Shi , et al., “80 Years of Extracellular Vesicles: From Discovery to Clinical Translation,” Extracellular Vesicles and Circulating Nucleic Acids 7, no. 1 (2026): 165–233. 10.20517/evcna.2025.161 PMC12902917 41695594

[55] Y. Chen , X. Cheng , C. Zhu , et al., “A Microsphere‐Vesicle Hybrid System for Tumor Treatment Through Enhancement of Innate and Adaptive Immune Responses,” Biomaterials 325 (2026): 123587, 10.1016/j.biomaterials.2025.123587. 40763579

