# Supplementary material for: Bioengineered Stroma‐Camouflaged Nanovesicles Disrupt the IDO1/CD276 Immunosuppressive Circuit to Reinvigorate Antitumor Immunity
Source: Adv Sci (Weinh). 2026 Oct 25:e78142. Online ahead of print. doi: 10.1002/advs.78142 (PMC13696784; doi:10.1002/advs.78142)
Supplement: Supplementary file 1 [file ADVS-9999-e78142-s001.docx]

**Bioengineered Stroma-Camouflaged Nanovesicles Disrupt the IDO1/CD276 Immunosuppressive Circuit to Reinvigorate Antitumor Immunity**

Ziqi Zhang, Jing Luo, Mengdi Zhang, Jingying Zhuang, Yating Zheng, Yang Zhang, Zhihong Huang, Xinhua Lin, Shuzhen Xu**^*^**, Hong Yao**^*^**, Gang Liu**^*^**, Bing Chen**^*^**

Z. Zhang, M. Zhang, J. Zhuang, X. Lin, S. Xu, H. Yao, and B. Chen

Key Laboratory of Nanomedical Technology (Education Department of Fujian Province), Department of Pharmaceutical Analysis, School of Pharmacy, Fujian Medical University, Fuzhou 350122, China

E-mail: BingChen_001@fjmu.edu.cn; yauhung@126.com

J. Luo

Department of Urology, Brigham and Women’s Hospital, Harvard Medical School, Boston, MA 02115, USA

Y. Zheng, Y. Zhang, G. Liu

State Key Laboratory of Molecular Vaccinology and Molecular Diagnostics & Center for Molecular Imaging and Translational Medicine, School of Public Health, Xiamen University, Xiamen 361102, China

E-mail: gangliu.cmitm@xmu.edu.cn

Z. Huang

Public Technology Service Center, Fujian Medical University, Fuzhou, Fujian Province 350122, China

S. Xu

Core Laboratory, Fuzhou Hospital of Traditional Chinese Medicine Affiliated to Fujian University of Traditional Chinese Medicine, Fuzhou, Fujian, China.

E-mail: shuzhenxu@stu.xmu.edu.cn

**Figure S1.** (A) Standard curve of DOX determined by HPLC. (B) Standard curve of BMS determined by HPLC.

**Figure S2.** The effect of different concentrations of IFN-γ on CD276 expression in 4T1 cells.

**Figure S3.** Quantitative analysis of the effect of different concentrations of IFN-γ (ng/mL) on IDO1 enzyme expression in 4T1 cells (n = 3).

**Figure S4.** Quantitative analysis of 4T1 cells treated with different materials for 24 hours using a flow cytometer (n = 3).

**Figure S5.** Effects of different treatment groups on the percentage of GzmB-positive cells within the CD8+ T-cell population in tumor tissue (n = 3).

**Figure S6.** Endpoint serum biochemical indicators of liver and kidney function in 4T1 tumor-bearing mice after different treatments: (A) ALT, (B) AST, (C) ALB, (D) γ-GT, (E) CREA, and (F) UA. Green dashed lines indicate the reference ranges shown in the original analysis (n = 3).

**Figure S7.** Quantification of CD25 immunohistochemical staining in tumor tissues.

**Figure S8.** (A) Quantification of CD8 fluorescence intensity in tumor tissues., (B) Quantification of CD38 fluorescence intensity in tumor tissues.
